# Evolution of a Signaling Nexus Constrained by Protein Interfaces and Conformational States

**DOI:** 10.1371/journal.pcbi.1000962

**Published:** 2010-10-14

**Authors:** Brenda R. S. Temple, Corbin D. Jones, Alan M. Jones

**Affiliations:** 1 R. L. Juliano Structural Bioinformatics Core Facility, University of North Carolina at Chapel Hill, Chapel Hill, North Carolina, United States of America; 2 Department of Biochemistry and Biophysics, University of North Carolina at Chapel Hill, Chapel Hill, North Carolina, United States of America; 3 Department of Biology, University of North Carolina at Chapel Hill, Chapel Hill, North Carolina, United States of America; 4 Carolina Center for Genome Sciences, University of North Carolina at Chapel Hill, Chapel Hill, North Carolina, United States of America; 5 Department of Pharmacology, University of North Carolina at Chapel Hill, Chapel Hill, North Carolina, United States of America; Baylor College of Medicine, United States of America

## Abstract

Heterotrimeric G proteins act as the physical nexus between numerous receptors that respond to extracellular signals and proteins that drive the cytoplasmic response. The Gα subunit of the G protein, in particular, is highly constrained due to its many interactions with proteins that control or react to its conformational state. Various organisms contain differing sets of Gα-interacting proteins, clearly indicating that shifts in sequence and associated Gα functionality were acquired over time. These numerous interactions constrained much of Gα evolution; yet Gα has diversified, through poorly understood processes, into several functionally specialized classes, each with a unique set of interacting proteins. Applying a synthetic sequence-based approach to mammalian Gα subunits, we established a set of seventy-five evolutionarily important class-distinctive residues, sites where a single Gα class is differentiated from the three other classes. We tested the hypothesis that shifts at these sites are important for class-specific functionality. Importantly, we mapped known and well-studied class-specific functionalities from all four mammalian classes to sixteen of our class-distinctive sites, validating the hypothesis. Our results show how unique functionality can evolve through the recruitment of residues that were ancestrally functional. We also studied acquisition of functionalities by following these evolutionarily important sites in non-mammalian organisms. Our results suggest that many class-distinctive sites were established early on in eukaryotic diversification and were critical for the establishment of new Gα classes, whereas others arose in punctuated bursts throughout metazoan evolution. These Gα class-distinctive residues are rational targets for future structural and functional studies.

## Author Summary

Proteins evolve new protein-protein interactions through changes to their residues. Many residue changes are harmful because they disrupt important existing interactions and functions. The more interactions a protein participates in, the more difficult it is to make changes that are not harmful to the protein. And yet, proteins with many existing interactions are also likely to evolve new functions or new interactions. How does evolution occur in the context of a well-constrained protein with many interactions? We studied the heterotrimeric G protein subunit Gα, a multi-functional protein that acts at the nexus between receptors responding to extracellular signals and the cytoplasmic proteins driving the response within the cell. The Gα subunit participates in numerous interactions that have constrained much of Gα evolution; yet Gα has diversified into four functionally specialized classes. We developed an approach that identifies key residue changes important to the evolution of Gα functionality and class, and gained insight into the types of residue changes that occurred both early and late in the evolution of Gα function. By studying these critical residues in Gα we can de-couple the many functionalities of this signaling nexus.

## Introduction

How is functional novelty generated when a protein is highly constrained by its many interactions with other proteins and by its critical role in the cell? In these proteins, new mutations are likely to have deleterious consequences by disrupting some important function within the cell due to the high probability that the mutation interferes with at least one of the many interactions. The Gα subunit of the heterotrimeric G protein complex is a classic example of a highly constrained family of proteins. Heterotrimeric guanine nucleotide binding proteins (G proteins) serve as physical couplers between cell surface 7 transmembrane (7TM) G-protein coupled receptors (GPCRs) and downstream targets known as effectors. As such, they are critical for signal transduction in eukaryotes and act as a nexus of extracellular signaling and intracellular changes. The Gα subunit, therefore, is ideal for understanding how functional novelty arises when a protein that is highly constrained evolves.

**Figure 1:**
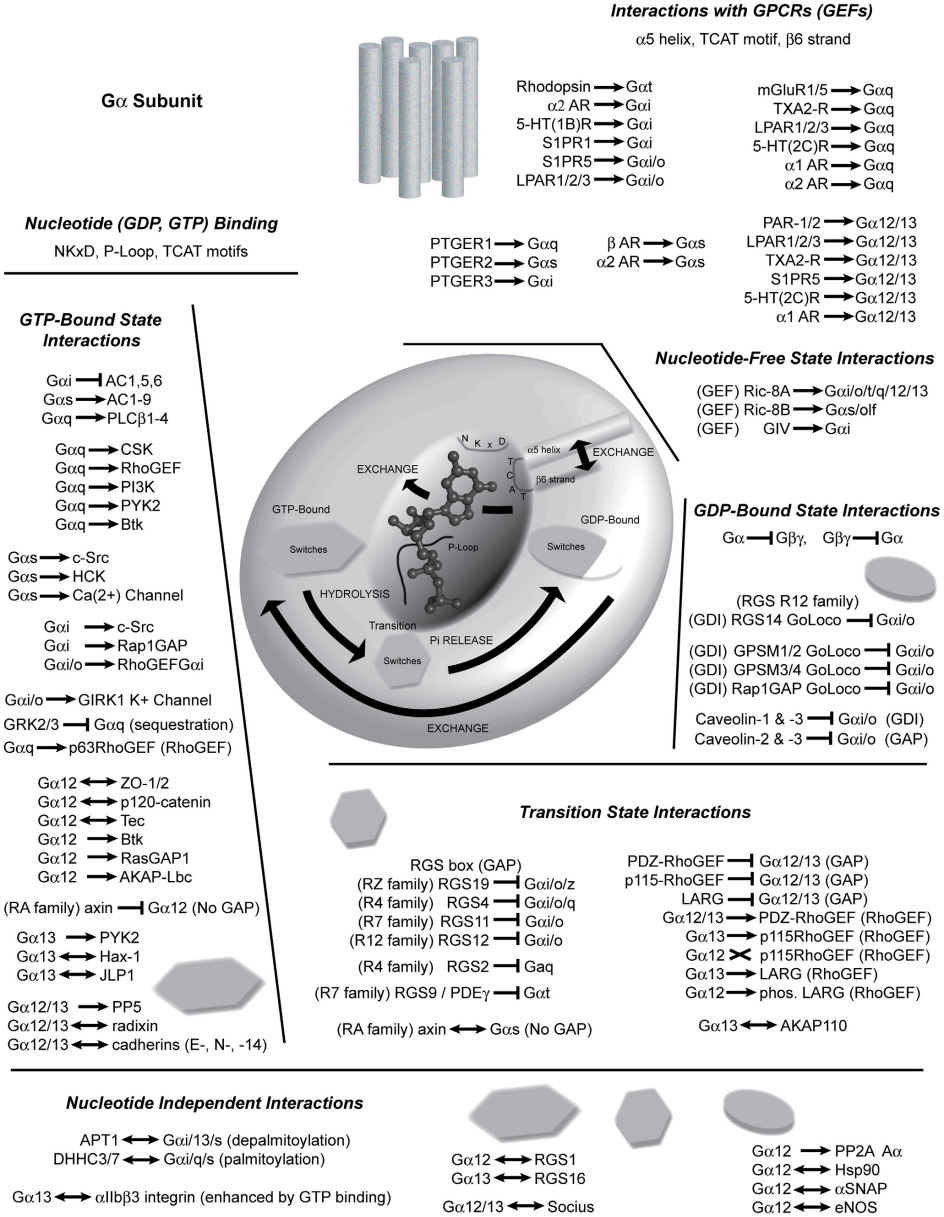
Gα as a regulated molecular signaling nexus. This graphic of the Gα signaling nexus delineates functional elements within the molecule such as nucleotide binding (e.g. NKxD motif, TCAT motif, P-loop) and GPCR-driven nucleotide exchange (α-helix 5, β-strand 6), the different conformations of Gα (i.e. transition and GDP-, GTP-bound states), along with mammalian macromolecules that have been reported to directly interact with Gα. Reported interactions are classified by nucleotide dependence and by functional outcome (GDI, GEF, etc.). The single-headed arrow represents an interaction leading to signaling, the ‘X’ represents an interaction that does not lead to signaling, a blunt arrow represents interactions leading to signal termination, while the double-headed arrow represents a neutral physical interaction. While the list of reported interactions is intended to be extensive, it is not intended to be exhaustive, particularly in regard to the GPCRs. An expanded figure legend with additional references is in Text S1.

G proteins have three subunits – Gα, Gβ and Gγ. In humans, there are 21 Gα, 6 Gβ and 12 Gγ subunits, which can be combined into many possible heterotrimers [1]. The human G protein signaling pathway is diverse and complex with approximately 850 GPCRs and dozens of G protein effectors (Jones and Assmann, 2004). These complex interactions mean that changing any residue of a G protein may have profound pleiotropic effects (Figure 1). For example, a promiscuous Gα subunit may interact with dozens of receptors and effectors [2], thus any mutation resulting in novel receptor or effector interactions potentially impacts many signaling pathways and can disrupt other interactions such as heterotrimer formation. The Gα subunit also has endogenous enzymatic activity, GTP hydrolysis, which puts further mechanistic constraints on the protein structure, and also drives a functional role where Gα acts as a “timer” with the intrinsic and regulated hydrolysis activity controlling the length of time the signaling pathways are activated as well as the amplitude of the response. Gα subunits are further constrained because they must cycle through multiple conformational states; any alteration of these states can disrupt the function of the G-protein and its interactions. The Gα structural core contains nucleotide-binding domains and switches that establish the basal, active and transition-state conformations. All Gα subunits bind GDP and GTP within a nucleotide pocket comprised of structural elements called a P-loop and an NKxD motif (Figure 1, center). The basal state occurs when Gα is bound to GDP, driving switch conformations compatible with interactions to Gβγ subunits. Nucleotide exchange of GDP for GTP generates switch conformations that define the active state and form an interface for target downstream effectors while driving the dissociation of Gα from Gβγ. The transition state for nucleotide hydrolysis is a third conformational state which is only recognized by a subset of interactors involved in regulation of G protein signaling. The Gα amino terminus, which is defined by an extended helix that affects associations with the Gβγ dimer, is involved in delimiting the subunit to the membrane through covalent attachment to lipids, and associates with a GPCR. Certain residues at both the amino and carboxyl termini of Gα interact with the activated receptor and are involved in nucleotide exchange [3], [4].

Besides receptors and effectors, a great many other proteins interact with the Gα subunit to control the activation state (Figure 1). Gα subunits are regulated by molecules that control its activation by acting as guanine-nucleotide exchange factors (GEFs) and guanine-nucleotide dissociation inhibitors (GDIs), and its deactivation by acting as GTPase activating proteins (GAPs). Thus, the surface of Gα evolved multiple, specific protein-protein interaction interfaces – such as those for the Gβγ dimer, the receptor, and the cognate effectors or regulators – many of which were partially or completely overlapping. The complexity of the Gα surface means that pleiotropic effects would most likely accompany any single mutation.

**Figure 2:**
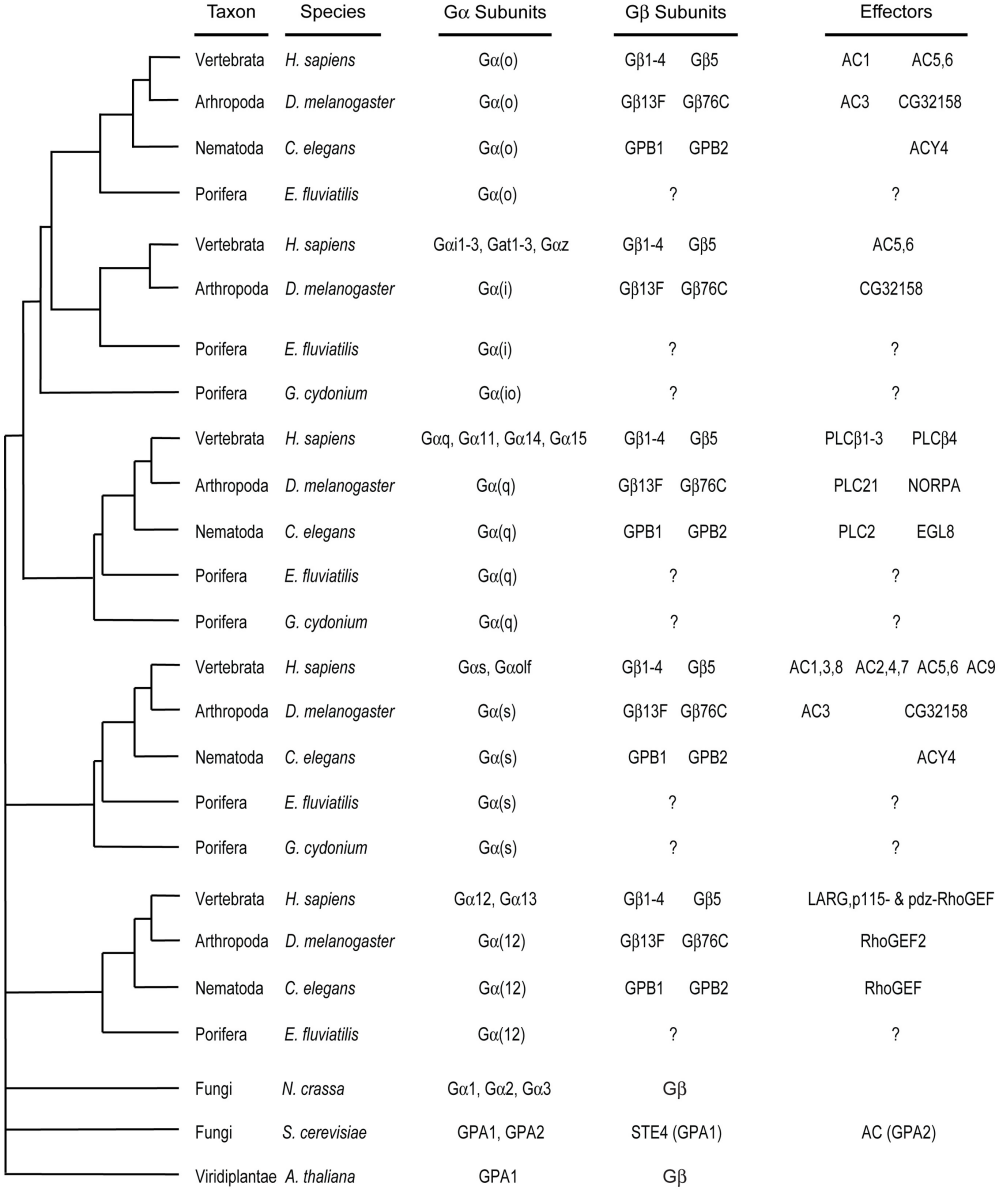
Evolution of the molecular signaling nexus Gα. A heuristic tree that captures commonly accepted characteristics between taxa and Gα and Gβ subunits from representative genomes is shown to the left. Plants have a single Gα subunit and the two fungi have either 2 or 3 Gα that do not fit any of the 4 animal subtypes. Thus, the evolution of the Gα subunit from a single gene to the multigene family evident in mammals occurs within eukaryotes. Divergent Gα subunits found in some genomes are not included in this diagram. Homologs of well-studied proteins that interact with mammalian Gα subunits are indicated to the right. For incomplete genomes, the presence of the interactor may be indeterminate and is indicated by a ‘?’. Homology indicates the presence of a protein in the given organism, but not all interactions have been verified in lower metazoans. As is evident from the chart, plant Gα subunits contain a single known interacting protein, the Gβγ heterodimer. The number of interacting proteins grew steadily throughout evolution of the GPCR signaling system. No bootstrap or other credibility scores are shown for the heuristic tree as this is not intended to be a definitive phylogeny. Marine sponge = *Geodia cydonium*; Freshwater sponge = *Ephydatia fluviatilis*; Worm = *Caenorhabditis elegans*; Fruitfly = *Drosophila melanogaster*; Human = *Homo sapiens*.

Given these enormous constraints, how did Gα evolve from a single ancestral subunit to form the four main classes in humans (G(io), G(q), G(s), G(12)) with multiple subtypes (Figure 2), each with distinguishing sets of sub-functionalities? Gene duplication clearly provided the raw genetic material, but how these nascent duplicates acquired class-distinctive functionality is unclear [5]. A confounding factor is the sporadic emergence of interacting proteins throughout evolution (Figure 2). Three new developments enabled us to answer these questions. First, plants, in contrast to animals, have a greatly simplified G-protein signaling pathway [6] thus providing a working structure of an ancestral-like Gα subunit. Second, there is now a wealth of comparative genomic sequence data to track over evolutionary time how and when new functionality was added to the ancestral Gα subunit [7], [8]. Third, there are now atomic structures of Gα in three conformational states, in its heterotrimeric complex, as well as co-resolved with several different interacting proteins. These structures allow us to place the evolutionary changes that we observe into a spatial context. These spatial data then reveal which protein interfaces or conformational states provide the evolutionary pressure driving the emergence of class-distinctive amino acid changes.

To understand how the functional diversity of extant Gα subunits arose from a single ancestral core, we need to know how the intermolecular interactions of this signaling network constrained the evolution of structure to a set of core sub-functions associated with all the subunits, and how differentiated structural elements drove the emergence of unique sets of sub-functions within subgroups of Gα subunits. Typically, this type of analysis begins with a deduction of the ancestral structure along with ancestral core functionalities, followed by an analysis of retained modifications as subunits duplicated and diverged throughout class evolution [9]–[11]. This approach by itself is recalcitrant to dissecting structure-function relations in a signaling nexus like Gα because this large Gα family has members containing both partially overlapping and non-overlapping protein-protein interaction interfaces as well as multiple distinct conformations. For example, many interfaces are dependent on the nucleotide-bound state.

We developed a broadly-applicable, synthetic approach for identifying key functional sites in Gα using structural data from mammalian Gα subunits and sequence data from across the diversity of eukaryotes. We used mutual information theory [12], [13] to select functional sites and phylogenetic analyses to show when and how the ancestral Gα diverged. Mutual information theory is a statistically-robust method for identifying the subset of sites most critical to the preservation of the functional core of Gα and those evolved sites important to diversification among subclasses of Gα (class-distinctive sites). We used this strategy to select evolutionarily important sites, setting criteria to automatically select sites that are uniquely associated with the functional divergence of a single Gα class and therefore likely arose from modifications to parental functionality after gene duplications. We used the atomic structures of Gα complexes to place our class-distinctive sites in a three-dimensional context and to identify sources of constraints on certain class-distinctive sites. We traced changes in these class-distinctive sites over evolutionary time to show when and how each of these functional Gα classes emerged.

The initial impetus was to determine the structural requisites for Gα class-specific functionality to enable regulation of activation/deactivation, coupling, and specificities. However, given the broad and deep genomic resources available, the approach described here is applicable to any protein that is a member of a gene family that underwent divergence through multiple, closely-spaced gene duplications, such as phospholipase C proteins, kinases, GPCRs, etc. Our analysis yielded several surprising results regarding Gα. For example, class-distinctiveness within the functional core was conferred by relatively few sites per class. A closer look at these sites within a class revealed unique features, functions, and interfaces of that class. Class-distinctive sites were found to impact all Gα classes and functionalities in addition to protein-protein interactions, such as the nucleotide binding properties that control signaling pathway dynamics. We used these data to propose explanations for several intriguing questions about Gα functional divergence and to propose sites that are rational targets for generating class specific mutations.

## Results

### Mutual information theory identified key residues underlying the functional diversification of Gα classes

We applied mutual information theory to 14 of the 17 vertebrate Gα subtypes listed in Table S1 to identify class-distinctive sites that contribute to functional differences among subgroups of Gα subunits. A robust multiple sequence alignment (MSA) (Text S2) was achieved by seeking consensus from sequence alignments generated by different MSA programs and also by structural comparisons of different Gα gene family members (see Materials and Methods).

**Figure 3:**
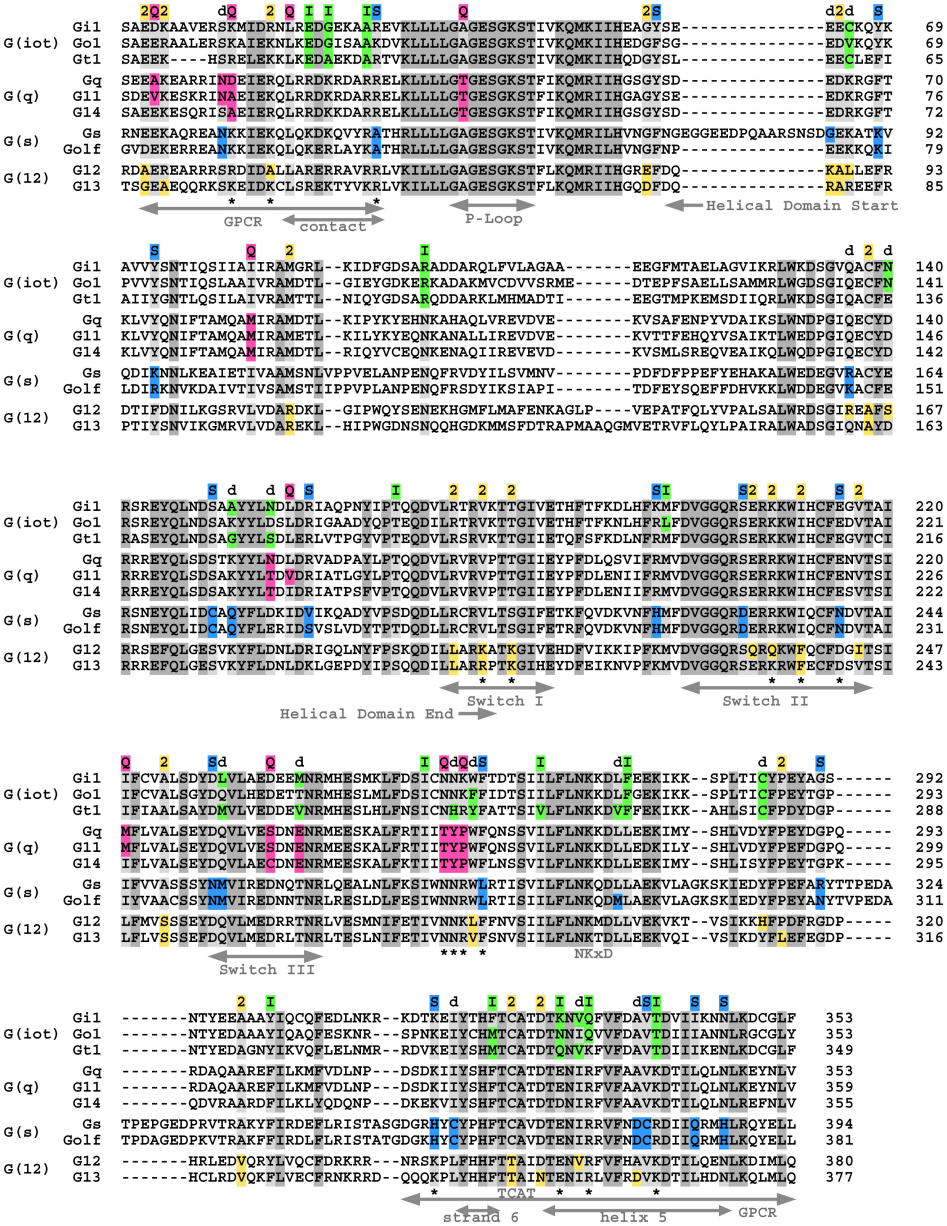
Alignment of select human Gα subtypes highlighting invariant and class-distinctive sites. Invariant residues are conserved across all 4 Gα classes (INV: colored dark gray) while class-distinctive sites are conserved across 3 of the 4 Gα classes to a non-distinctive (**η**: colored light gray) amino acid value. At class-distinctive sites, distinctive (**∂**) amino acid values are allowed in the remaining class but are not required to be absolutely conserved within all sequences in the distinctive Gα class, thus allowing for subclass variation at that site. Some sites are identified as class-distinctive based on variation in a single non-human sequence. See Table S2 to identify sequences where **∂** occurs. **d** sites lie within 5 Å of a distinctive site but are conserved in 2 classes (see Table S2 and Table S3 for summaries). **∂** amino acid values are colored according to Gα class and noted above the alignment: ‘I’ = G(io) site (green); ‘Q’ = G(q) site (magenta); ‘S’ = G(s) site (blue); ‘2’ = G(12) site (yellow orange) and ‘d’ = **d** site. Functional regions are indicated below the alignment, including regions important for coupling to the receptor (GPCR), guanine-nucleotide-dependent conformational change (switches I, II, III) and nucleotide binding (P-loop, NKxD, TCAT). Also noted below the alignment (‘*’) are distinctive sites discussed in more detail in results. Distinctive sites for all 4 Gα classes were defined using 58 mammalian Gα sequences from 14 subtypes and a reduced amino acid alphabet (Materials and Methods).

We identified 106 invariant and 59 class-distinctive sites from an MSA of 58 mammalian Gα sequences encompassing all 4 major classes (Figure 3). Our criterion for labeling a site in the alignment as class-distinctive was that it had an invariant amino acid value in sequences of three of the Gα classes and a different amino acid value in sequences from the fourth Gα class (as defined using a reduced amino acid alphabet, see Materials and Methods). This criterion limited the analysis to those sites that reflect a modification within a single, given class of a parental sub-functionality after a gene duplication event. Our more restrictive criteria, as compared to the earlier sequence-based analyses on the Gα family [14], [15], allowed us to immediately hypothesize that each class-distinctive site contributed to a unique functionality of the given Gα class. A corollary is that any Gα class for which class-distinctive sites could not be identified would imply a Gα class that had conserved parental functionality without modification. This was not the case for mammalian Gα as we found class-distinctive sites for all 4 classes (14 G(io), 10 G(q), 16 G(s), and 19 G(12) sites; these sites are labeled with an ‘I’, ‘Q’, ‘S’, and ‘2’, respectively, in Figure 3). The distinct amino acid value (designated **∂** – distinct) was not required to be absolutely conserved within all sequences in the distinctive Gα class, thus allowing for sub-class variation at that site. In our initial analyses, we defined the conserved amino acid value (designated **η** – not distinct) to be invariant among all sequences in the remaining 3 classes—implying that these **η** amino acids were functionally constrained in the ancestor. Sites with different evolutionary histories are apparent in Figure 3. Some sites have a single **∂** amino acid value for all sequences within a class, or distinctive values in only a subclass or even in just a single sequence. At some sites, however, there is more than one **∂** amino acid value, implying subclass variation. There was no penalty placed on the occurrence of **η** amino acids within the distinctive class, allowing a site to be ancestral-like early in the evolution of a given Gα class but then later acquiring class-distinctness. Table S2 summarizes the class-distinctive sites, their **η** and **∂** residues, and their evolutionary histories. The class-distinctive sites are displayed on the Gα_i1_ **•**Gβγ heterotrimeric complex structure in Figure S1 in both space-filling and cartoon rendering for relative positioning of the distinctive sites from different classes. (A comparison between the sites identified here and the evolutionarily important sites identified by a different method – Evolutionary Trace – is presented in Text S3).

Class distinctiveness at a few positions was not readily apparent because of the stringency of the **η** residue criterion. A case in point – the residue located at position Y261 in human Gα_q_, a residue flanked by two G(q)-class distinctive residues (“TYP” in Gα_q_ in 4^th^ row of alignment in Figure 3); this begs the question of why this position was not originally classified as G(q)-distinctive. This is because one subtype in the G(io) class, namely Gα_t1_, has an amino acid value of H instead of the **η** value of N, thus precluding designation as class-distinctive using the given stringent criteria. To optimize the utility of the class-distinctive sites, we looked for neighboring residues that would contribute specificity to the new sub-functionality gained with the class-distinctive site, but which may have also independently diverged in a second class and would not, therefore, have been identified in our first analysis. These sites are analogous to position Y261 in Gα_q_. We used a contact distance of less than 5 Å between the two residues in the active state crystal structure to define which sites were neighbor to a given class-distinctive site. We looked for variation in sequences in the distinct class to select neighboring sites that likely contributed to the same specificity associated with the class-distinctive site. We limited variation to within one additional class, otherwise it was impossible to confidently assign one residue as the **η** residue. In this second level of scrutiny, we identified 16 more class-distinctive sites (designated **d** in Figure 3). We summarize these **d** sites in Table S3 and indicate the neighboring **∂** class-distinctive sites that flagged the second round of analysis. The core residues we identified, including invariant (106), **∂** (59) and **d** (16) class-distinctive sites, encompass approximately half (46–52%) of the total number of residues in the Gα subunit.

### Class-distinctive sites lie within regions of known important functionality

Functional regions are enriched with both unchanging core residues and evolving class-distinctive sites (Figure 3); regions that are less critical for the known functionality of a particular subtype typically lack class-distinctive sites. For example, residues at both termini that comprise the GPCR coupling interface are enriched with class-distinctive sites (20 **∂** class-distinctive sites [plus 4 **d** sites] of 66 residues). Similarly, switches I, II, and III are also enriched for **∂** and **d** class-distinctive sites (11 [plus 2] of 44). Three of the most functionally important regions of a Gα subunit are the GPCR binding interface, the α5 helix with its β sheet enclosure, and the 3 switches. The GPCR, through its interactions with the Gα subunit, determines which extracellular signal is being received and which pathway will be stimulated by that signal. The α5 helix and surrounding residues are critical for receptor-mediated nucleotide exchange. The switches are critical for interactions with target effectors, GEFs, GAPs and GDIs that affect the response and state of the Gα. In most cases, changes at these sites are deleterious. The evolutionary shifts at these critical sites, however, suggest a fundamental alteration in the function for that Gα class.

### Classes diverged by modifying Gα coupling to the GPCR and, in some cases, by modifying the switches

After duplication, each Gα gene diverged by evolving class-distinctive sites in a subset of – but not all –functionally-important regions (Figure 3). Since a different subset of functional regions were modified within each gene subfamily, the set of regions selected for evolving class-distinct functionality become characteristic for that gene or gene subfamily. For example, G(12) is unique in that it has class-distinctive sites in switch I. In contrast, both G(s) and G(12) have distinctive sites in switch II. Switches I and II are involved in binding the Gβγ heterodimer [16], [17] and other proteins such as regulators of G protein signaling (RGS) that stimulate Gα GTPase activity [18], [19]. A change in a switch commonly adds or removes critical contacts between the Gα and its effectors or regulators, suggesting that the changes in switch I of G(12) altered the interactions between G(12) and some of its binding partners, potentially regulatory proteins (see below). Analogously, class-distinctive sites evolved in switch III and the upstream region in G(q) and G(s), with G(io) containing **d** sites that also have **∂** residues in G(q) or G(s) subunits. However, there are no G(12)-distinctive sites located in switch III. Switch II (helix 2) and the region upstream of switch III (helix 3 and loop) is an interface for cognate effectors [20], again suggesting altered interactions in these Gα classes, but with effectors this time, allowing for the partially overlapping interfaces of regulatory and effector proteins on the Gα subunit. The GPCR coupling region, distributed over both termini, contains class-distinctive residues from all 4 major classes. These differing patterns of class-distinctive sites between switches and the GPCR interaction region are consistent with the coupled receptor and effector class specificity noted by Lichtarge et al. [14], but also indicate that natural selection exploited Gα as an existing signaling nexus by independently modifying individual regions associated with sub-functionalities (such as switch I) so that new connections in the network between effectors, regulators and receptors formed. These changes to different subsets of functionally-important regions within the Gα subunit ultimately resulted in new proteins with altered function and in new class-specific signaling pathways.

Gα subunits are not mere scaffolds for protein-protein interactions; they also affect signaling dynamics. We propose that control of GPCR based signaling pathways occurs through sequence-based modifications to Gα that indirectly affect nucleotide binding by directly affecting interactions with regulators such as GPCRs, GDIs, GAPs and GEFs. The α5 helical region, discussed below, has been shown to be important for receptor mediated exchange [21]. The α5 helical region contains class-distinctive sites from all classes except for G(q). Other regions are also involved in nucleotide binding. G(q) subunits are unique because they contain a class-distinctive site in the P-loop associated with nucleotide binding and because G(q) subunits have an undetectable basal nucleotide exchange rate [22]. Directly modifying nucleotide exchange properties within the different classes throughout evolution enhances the functional role of Gα as a “timer” controlling the length of time of activation of the different signaling pathways.

We hypothesize that the evolutionary patterns associated with a small set of class-distinctive sites within these largely autonomous functional domains of Gα predict residues that are critical for the functional specificity of these domains within each Gα class. In the following analyses, we test this hypothesis and link these evolutionary changes with class specific functions showing how this analytical framework can explain several conundrums regarding the structure and function of specific Gα subunits. We will explain (1) how functional specificity evolved, (2) how Gα subunits evolved class-specific functionality in their active state without affecting their inactive state, (3) how different but structurally-related Gα subunits evolved opposing functional outcomes, and (4) how new functionality evolved by modifications to residues participating in intramolecular interactions – versus intermolecular interactions – thereby controlling activation of the Gα subunit, and (5) how Gα diversified throughout metazoan evolution within and between functional classes.

### An example of the evolution of functional specificity: Two changes at G(q)-distinctive sites determine the specificity of the GRK2 interaction with Gα_q_

**Figure 4:**
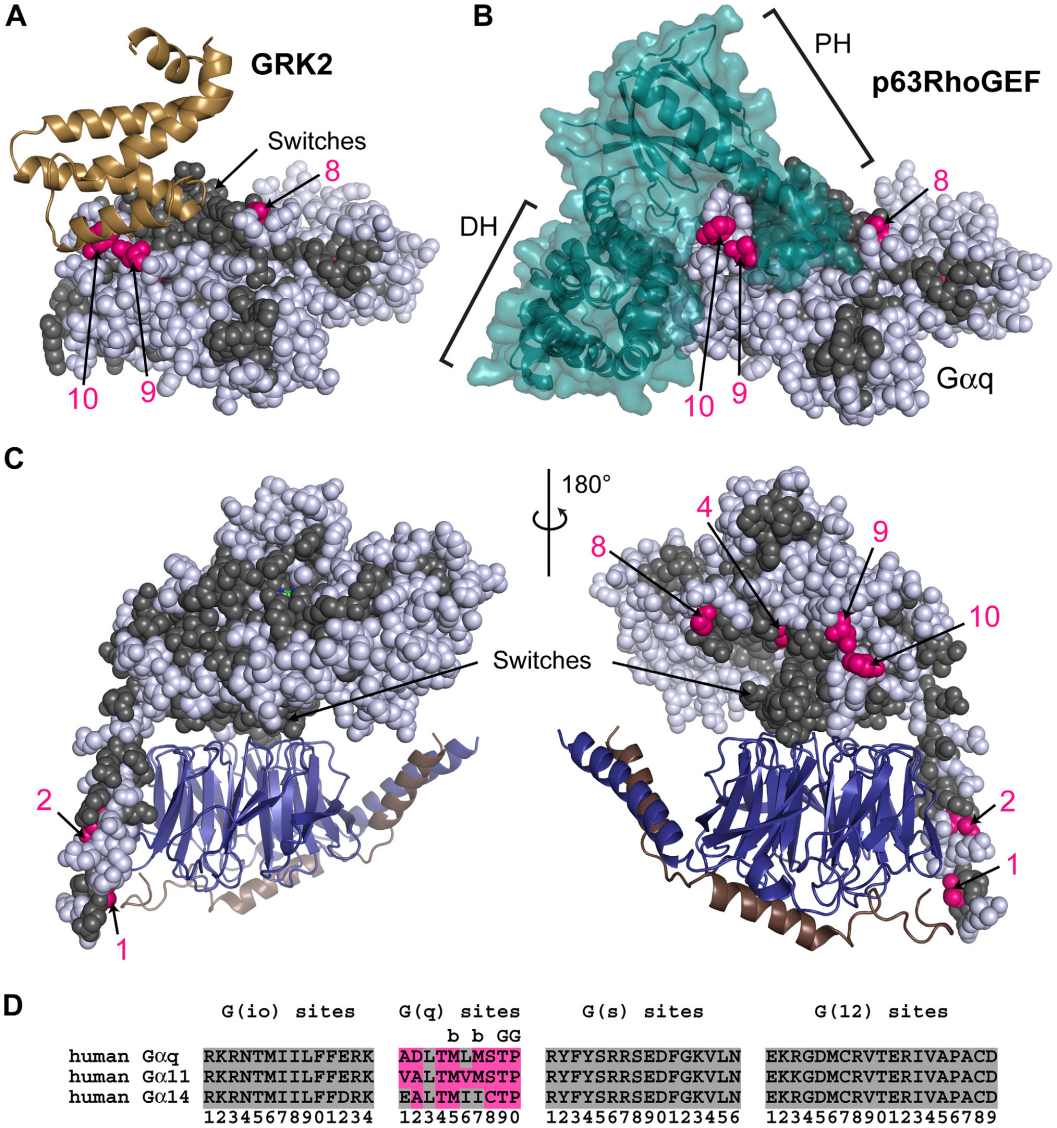
G(q) class-distinctive sites in structural context. (**A**) The RH domain of GRK2, shown as a sand colored cartoon display, in complex with activated Gα_i/q_ **•**GDP**•**Mg^2+^ **•**AlF_4_^−^ (PDB ID 2BCJ). In all structural panels in this figure, Gα_i/q_ is shown as spheres with core residues colored gray if the residues are conserved between Gα subunits (either INV (invariant) or **η** (non-distinct) amino acids) while G(q)-distinctive sites are colored hot pink only if they contain a **∂** (distinct) amino acid. Non-core residues and **d** sites are colored white. G(q)-distinctive sites are numbered according to their position in the signature sequence (see panel (D)). (**B**) The DH and PH domains of p63RhoGEF, in a teal colored cartoon and surface display, binds to activated Gα_i/q_ **•**GDP**•**Mg^2+^ **•**AlF_4_^−^ (PDB ID 2RGN). Gα_i/q_ is in the same orientation as panel A. (**C**) Homology model of Gα_q_ **•**GDP (sphere display) bound to Gβ**•**Gγ (deepblue/copper cartoon) heterodimer. Two orientations related by a 180° rotation about the vertical axis are shown. (**D**) Signature sequences are formed by grouping all distinctive sites for a given class together, removing all residues between individual distinctive sites of the noted class. The distinctive sites for each class are presented in order from the N-terminus to the C-terminus and numbered accordingly. Amino acids that correspond to the **∂** values at the G(q) site are colored hot pink. Sites that interact with GRK2 are denoted by ‘G’ above the site, while sites that are buried and not visible are denoted by ‘b’ above the site.

All three G(q) subtypes included in our study, Gα_q_, Gα_11_, Gα_14_, acquired two G(q)-distinctive sites that we propose are key to determining the specificity of the interaction with G protein-coupled receptor kinase 2 (GRK2). GRK2 inhibits GPCR signaling by phosphorylating activated GPCRs [23], and also by sequestering Gβγ and G(q) subunits through its pleckstrin homology (PH) [24] and RGS homology (RH) [25] domains, respectively. The atomic structure of an activated Gα_i/q_ chimera and Gβγ in complex with GRK2 [26] revealed the structural elements by which G(q) subunits are sequestered. In this complex, the RH domain of GRK2 interacted with switch II and an adjacent helix in Gα_i/q_ while the N-terminal helix of Gα_i/q_ – the domain inherited from Gα_i1_ – was disordered (Figure 4A). G(q) family subunits have no distinctive residues in switch II to distinguish this family from members of the G(io) class. However, G(q) family subunits do have two G(q)-distinct residues in the helix bordering switch II that formed part of the interface. Gα_q_ residue T260, labeled as the 9^th^ G(q)-distinctive site in Figures 4A and 4D, formed a hydrogen bond with a GRK2 residue in the structure. P262, G(q) site 10, was found to pack into a hydrophobic pocket formed by GRK2 and Gα_q_ residues. Tesmer et al. [26] reported that GRK2 binding to G(q) subunits was eliminated with a P262K mutation, which corresponded to a **∂** to **η** mutation at G(q)-distinctive site 10, and identified residues 261–263 as a specificity determinant region [26]. Residue Y261 was discussed earlier and is a **d** site with **∂** residues in both G(q) family members and in Gα_t1_ of the G(io) family. The role of G(q)-distinctive site 9 (T260) in contributing to specificity determination has not previously been recognized or verified experimentally.

We propose that G(q)-distinctive sites evolved to drive specificity of G(q) interactions to GRK2 but not p63RhoGEF, a G(q) specific effector that activates the small GTPase RhoA [27]–[29]. The atomic structure of p63RhoGEF complexed with activated Gα_i/q_ [30] revealed this interface contains no direct interactions with G(q)-distinctive residues (Figure 4B). In addition, the modeled heterotrimeric G-protein complex containing Gα_q_ (Figure 4C) revealed a parental interface on Gα_q_ for the Gβγ heterodimer. At present, only the Gα_q_ **•**GRK2 interface appears to constrain G(q)-distinctive sites 9 and 10.

### An example of the evolution of active-state-specific functionality: G(12)-distinctive residues in the switches are critical for the interaction between p115RhoGEF and Gα_13_ in the active state but do not disrupt the primordial interaction with Gβγ in the inactive state

**Figure 5:**
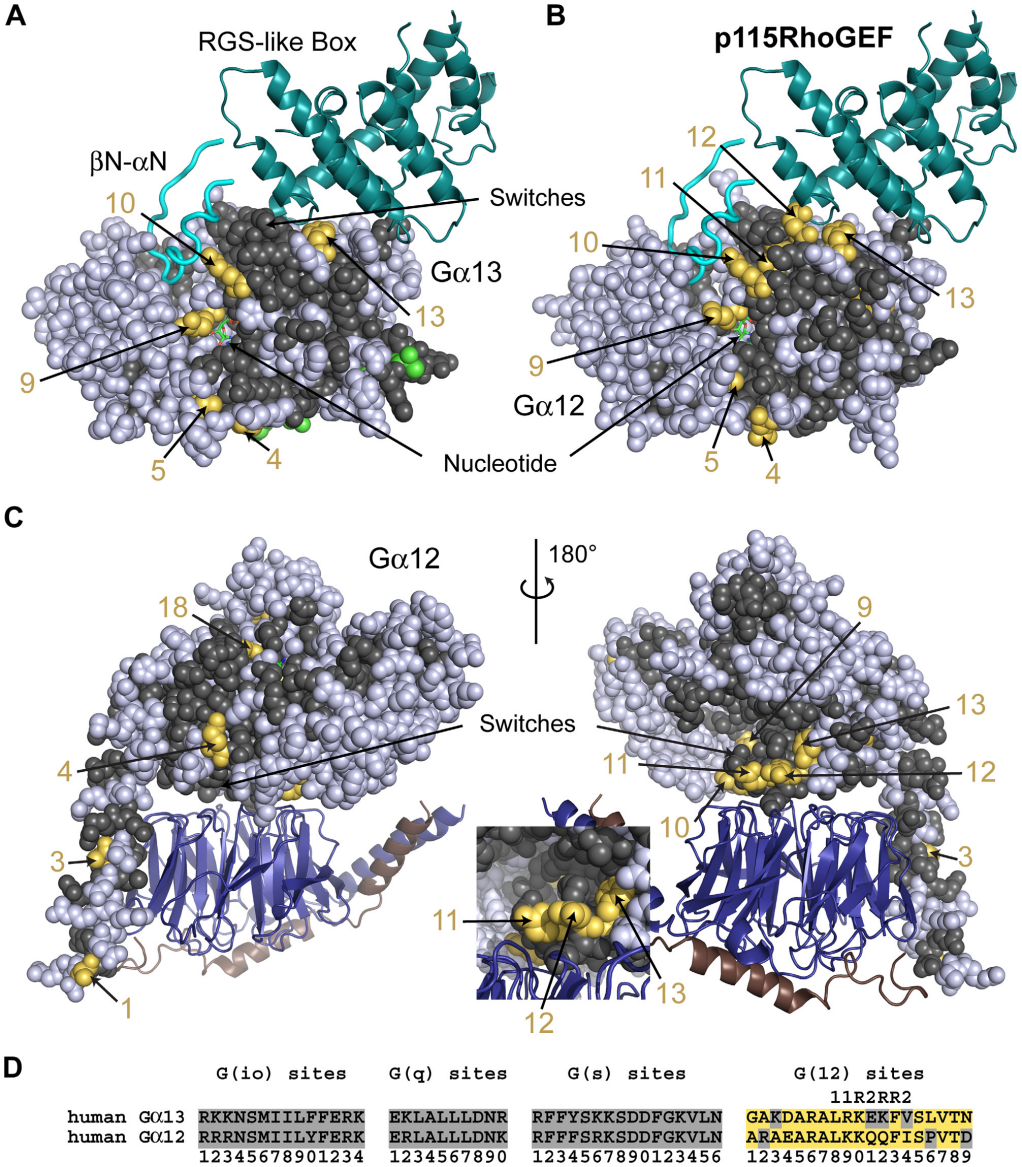
G(12) class-distinctive sites in structural context. (**A**) The structure of the p115RhoGEF RGS-like box domain (dark teal cartoon) and a βN-αN hairpin element (cyan loop cartoon) bound to an activated Gα_13/i1_ chimera (Gα_13/i1_ **•**GDP**•**Mg^2+^ **•**AlF_4_^−^) (PDB ID 1SHZ). In all structural panels in this figure, Gα is shown as spheres with core residues colored gray if they are conserved between Gα subunits (either INV (invariant) or **η** (non-distinct) amino acids) while G(12)-distinctive sites are colored yellow orange only if they contain a **∂** (distinct) amino acid. The chimeric Gα subunit in this structure also contained **∂** (distinct) amino acids at several G(io)-distinctive sites (green spheres). Non-core residues and **d** sites are colored white. G(12)-distinctive sites are numbered according to their position in the signature sequence (see panel (D)). (**B**) Model of Gα_12/i1_ in complex with p115RhoGEF. Gα is in the same orientation as panel A. (**C**) Homology model of Gα_12_ **•**GDP (sphere display) bound to Gβ**•**Gγ (deep blue/copper cartoon) heterodimer. Two orientations related by a 180° rotation about the vertical axis are shown. The inset is a close up view of the Gβγ binding region in the right view. (**D**) Signature sequences are formed by grouping all distinctive sites for a given class together, removing all residues between individual distinctive sites of the noted class. The distinctive sites for each class are presented in order from the N-terminus to the C-terminus and numbered accordingly. Amino acids that correspond to the **∂** values at the G(12) site are colored yellow orange. Sites that have direct interactions with p115RhoGEF are denoted by ‘R’ above the site, while additional sites in switches I or II are denoted by ‘1’ or ‘2’, respectively, above the site.

The Gα_12/13_•p115RhoGEF interface is dense with G(12)-distinctive sites (Figures 5A, 5B). Class-distinctive sites, analogous to those in the interaction of Gα_q_ with GRK2, contribute significantly to the specificity of interactions between the G(12) subunit family and p115RhoGEF. The G(12)-distinctive sites, however, lie in the switches, which are regions sensitive to the bound nucleotide. In contrast, the G(q)-distinctive sites driving the Gα_q_ specificity lie in a helix neighboring switch II, a region not sensitive to the state of the bound nucleotide. We hypothesize that the G(12)-distinctive sites confer effector and regulator specificity in the active and transition states (Gα_13_ in Figure 5A and Gα_12_ in Figure 5B), yet do not disrupt interactions with Gβγ in the inactive state (Gα_12_ in Figure 5C) even though the sites are in switches I and II, regions important for binding both Gβγ and p115RhoGEF.

The G(12) story is complicated by significant differences in the functional outcomes that result when the two different vertebrate G(12) subunits interact with p115RhoGEF. Specifically, Gα_13_, but not Gα_12_, activates RhoA when in complex with p115RhoGEF. Several of the G(12)-distinctive sites in switch II, which form part of the interface, show subtype variation within the gene family. This subtype-specific variation at G(12)-distinctive sites in switch II may contribute to this G(12) subtype difference in effector functional outcome (below).

P115RhoGEF is a G(12) specific effector that binds members of the G(12) family in a nucleotide-dependent manner and acts as a GAP toward Gα_12_ and Gα_13_ [31], [32]. P115RhoGEF also stimulates GEF activity on Rho GTPase when bound to Gα_13_, activation exerted via its DH and PH domains [31], [33]. The structure of the N-terminal domains of p115RhoGEF bound to an activated Gα_13/i1_ chimera (Figure 5A) suggested the GAP activity was associated with an N-terminal βN-αN hairpin element that was conformationally distinct from canonical RGS domains, which had also been shown to possess GAP activity toward Gα proteins [20]. Mapping our class-distinctive sites onto the structure of the Gα_13/i1_ **•**p115RhoGEF complex revealed an interface covering switches I and II of the Gα_13_ subunit, a region that possesses 7 G(12)-distinctive sites within these two switches. One (site 10, Figures 5A and 5D) of three G(12)-distinctive sites in switch I made a direct contact to the βN-αN structural element of p115RhoGEF. Mutating the **∂** amino acid value at site 10 (K204) diminished binding of p115RhoGEF to Gα_13_ [34], [35], verifying the importance of this G(12) site in the evolution of G(12) functional specificity. Chen et al. [20] also noted that R201, which is the **∂** amino acid in G(12)-distinctive site 9, acted as a tether between switch I and a Gα_13_-unique helical insert within the α-helical domain, suggesting that some distinctive sites may be important for switch conformation and intra-domain contacts rather than direct interactions at an interface.

The Gα_13/i1_ **•**p115RhoGEF structure revealed that the RGS-like box of p115RhoGEF bound to the Gα effector interface (switch II) rather than the typical regulator interface of Gα_13_. Chen et al. [20] proposed that, based on the effector-like interactions between switch II and the RGS-like box, Gα_13_ may act indirectly on the DH and PH domains of p115RhoGEF through the RGS-like box to exert the GEF activity on RhoA. We show that two (sites 12 and 13) of three G(12)-distinctive sites in switch II made direct contacts to residues of the RGS-like box of p115RhoGEF. Distinctive sites 11 and 12 in switch II show subtype variation, with the **η** amino acids evident in Gα_13_ at these sites (Figure 5A) and the **∂** amino acids in a model of Gα_12_ [36] bound to p115RhoGEF (Figure 5B). P115RhoGEF also acted as a GAP toward Gα_12_ [32], but Gα_12_, unlike Gα_13_, did not mediate RhoA activation [31]. It is possible that the subclass sequence variation at these two sites account for this subtype specific loss of p115RhoGEF activity, but the story may be more complex (see Text S4 for additional discussion).

Although these G(12)-distinctive sites confer specificity to the interaction with p115RhoGEF when the Gα subunits are in the active/transition state (Gα_13_ in Figure 5A, Gα_12_ in Figure 5B), the G(12) subunits still bind Gβγ in the inactive state (Gα_12_ in Figure 5C). The switches in the Gα_12_ **•**GDP conformation form a ledge with Gβγ binding to the side of the ledge shaped by conserved residues (Figure 5C, right view and inset). The G(12)-distinctive residues (sites 10–13) are on the opposite side of the ledge, positioned away from the Gβγ interface, and thus do not disrupt G(12) family members binding to the Gβγ heterodimer. Gβγ is not the only macromolecule which binds the inactive conformation. GoLoco motifs found in several proteins also bind the Gα**•**GDP conformation (see below and also Figure 1), but GoLoco motifs bind in the concavity formed by the G(12) sites 10–13 and the main Gα structure (Figure 5C, right view and inset). Several of the G(12) **∂** residues in the switches are positioned to discriminate among molecules that utilize this surface (data not shown), emphasizing the pleiotropic effects that arise whenever shifts are made in a molecule highly constrained by so many interactions.

### An example of the evolution of opposing outcomes in structurally similar Gα: G(s)-distinctive sites may drive the conformational changes within the Gα interface affecting the interactions of Gα_s_ and Gα_i_ with adenylyl cyclase

**Figure 6:**
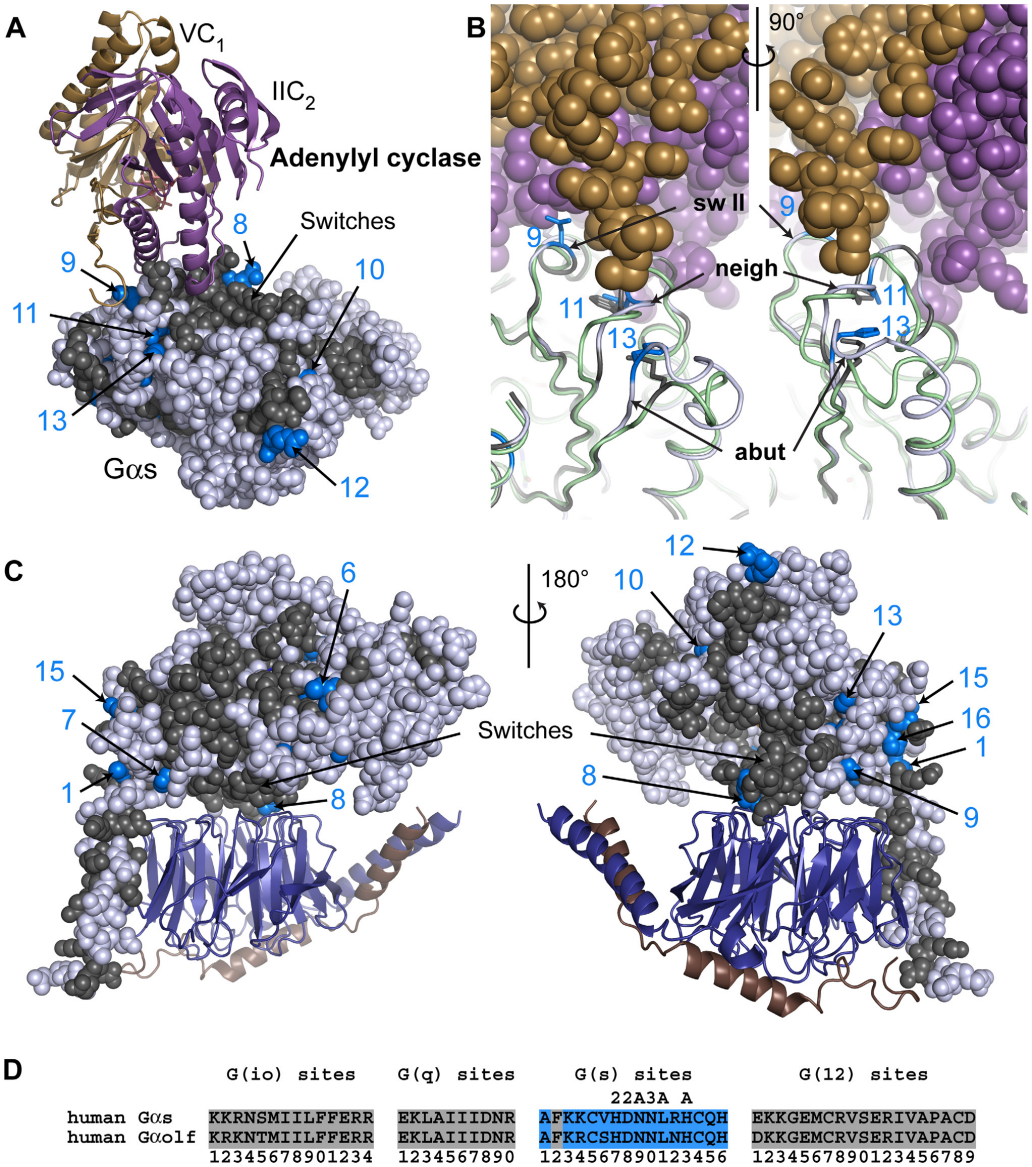
G(s) class-distinctive sites in structural context. (**A**) The structure of the catalytic domains of adenylyl cyclase (VC_1_ in sand cartoon, IIC_2_ in purple cartoon) bound to an activated Gα_s_ (Gα_s_ **•**GTPγS) (PDB ID 1AZS). In all structural panels in this figure, Gα_s_ is shown as spheres or cartoon with core residues colored gray if they are conserved between Gα subunits (either INV (invariant) or **η** (non-distinct) amino acids) while G(s)-distinctive sites are colored blue only if they contain a **∂** amino acid. Non-core residues and **d** sites are colored white. G(s)-distinctive sites are numbered according to their position in the signature sequence (see panel (D)). (**B**) Superimposition of Gα_s_ (light gray cartoon with **∂** amino acids at G(s) sites of interest rendered as blue sticks) and Gα_i1_ (PDB ID 1GIA in light green cartoon with corresponding **η** amino acids at G(s) sites in sticks and colored gray) highlighting sequence and backbone conformational changes in switch II (“sw II”) and loops near the adenylyl cyclase interface. The two views are related by a 90° rotation about the vertical axis. VC_1_ is in sand spheres and IIC_2_ is in purple spheres. G(s) site 11 lies in a loop neighboring switch II that forms part of the binding interface (“neigh”) while G(s) site 13 is in a loop that abuts the binding interface (“abut”). (**C**) Homology model of Gα_s_ **•**GDP (sphere display) bound to Gβ**•**Gγ (deepblue/copper cartoon) heterodimer. Two orientations related by a 180° rotation about the vertical axis are shown. (**D**) Signature sequences are formed by grouping all distinctive sites for a given class together, removing all residues between individual distinctive sites of the noted class. The distinctive sites for each class are presented in order from the N-terminus to the C-terminus and numbered accordingly. Amino acids that correspond to the **∂** values at the G(s) site are colored blue. Sites that have been proposed to be important to the interaction with adenylyl cyclase are denoted by ‘A’ above the site, while additional sites in switches II or III are denoted by ‘2’ or ‘3’, respectively, above the site.

Two Gα subunits interact with adenylyl cyclase (AC) with opposite functional outcomes. Gα_s_ stimulates AC, whereas Gα_i_ inhibits AC. Comparisons of the crystal structures of Gα_i1_ **•**GTPγS [37], [38] with those of Gα_s_ **•**GTPγS [39], and the Gα_s_ **•**GTPγS**•**VC_1_ **•**IIC_2_ **•**forskolin [40] complex prompted Sunahara et al. [39] to suggest that the interface on Gα_s_ for AC (Figure 6A), which is comprised of switch II (Figure 6B “sw II”) and its neighboring loop (Figure 6B “neigh”), was similar in sequence but dissimilar in shape to the same region on Gα_i1_ (Figure 6B; Gα_s_, gray cartoon; Gα_i1_, green cartoon). They concluded that disparately-shaped binding surfaces, not sequence differences, drove the distinct functional outcomes [39], [40]. With their model in mind, we noted three G(s)-distinctive sites (sites 7, 8, and 9 in Figures 6A and 6D) are in or near switch II. Site 9 is the only one of these sites that has a direct interaction with AC (Figure 6B), but a mutation of three residues at the interface that also included site 9 resulted in only a threefold reduction in AC activation [39], [41], consistent with the proposed role of conformational differences, not sequence differences, as the source of discrimination between Gα_i1_ and Gα_s_.

There are two other G(s)-distinctive sites near the interface: G(s) site 11 that lies in the neighboring loop that forms part of the interface, and site 13 that lies in a loop abutting the interface (Figure 6B “abut”). The **η** amino acid in Gα_i1_ at G(s) site 13 is a solvent-exposed lysine, whereas the **∂** amino acid in Gα_s_ at the same site is a buried histidine. Adjustments to the backbone in the abutting loop allow for these different side chain orientations (Figure 6B) in the two Gα subunits. The abutting loop is different in sequence and length between G(s) and G(io) family members, which contributes to the conformational differences in this loop between the two families [39]. In contrast, the loop neighboring switch II containing G(s)-distinctive site 11 is similar in sequence and length between G(s) and G(io) family members [39], except for the single G(s) class-distinctive site, even though it adopts slightly different conformations in the two family members. Conformational differences in this neighboring loop may be driven both by sequence changes at site 11 – the bulky phenylalanine (**η** amino acid) in Gα_i1_ is shifted in position from the leucine (**∂** amino acid) in Gα_s_ [39] – and by the conformational changes in the abutting loop. Thus, conformational differences in these two loops leading to the opposite functional outcomes between Gα_i1_ and Gα_s_ are potentially driven by the class-distinctive sites in G(s) subunits. Both G(s)-distinctive sites 11 and 13 were identified by Sunahara et al. [39] in a structural analysis as critical components driving structural differences, which is consistent with earlier mutational studies replacing entire loops in the two Gα families [41]. This analysis suggests that G(s)-distinctive sites could influence the conformational changes that affect the interactions of Gα subunits with AC.

### An example of new functionality in intramolecular interactions: G(io)-distinctive sites in a helix controlling activation of Gα by the GPCR

**Figure 7:**
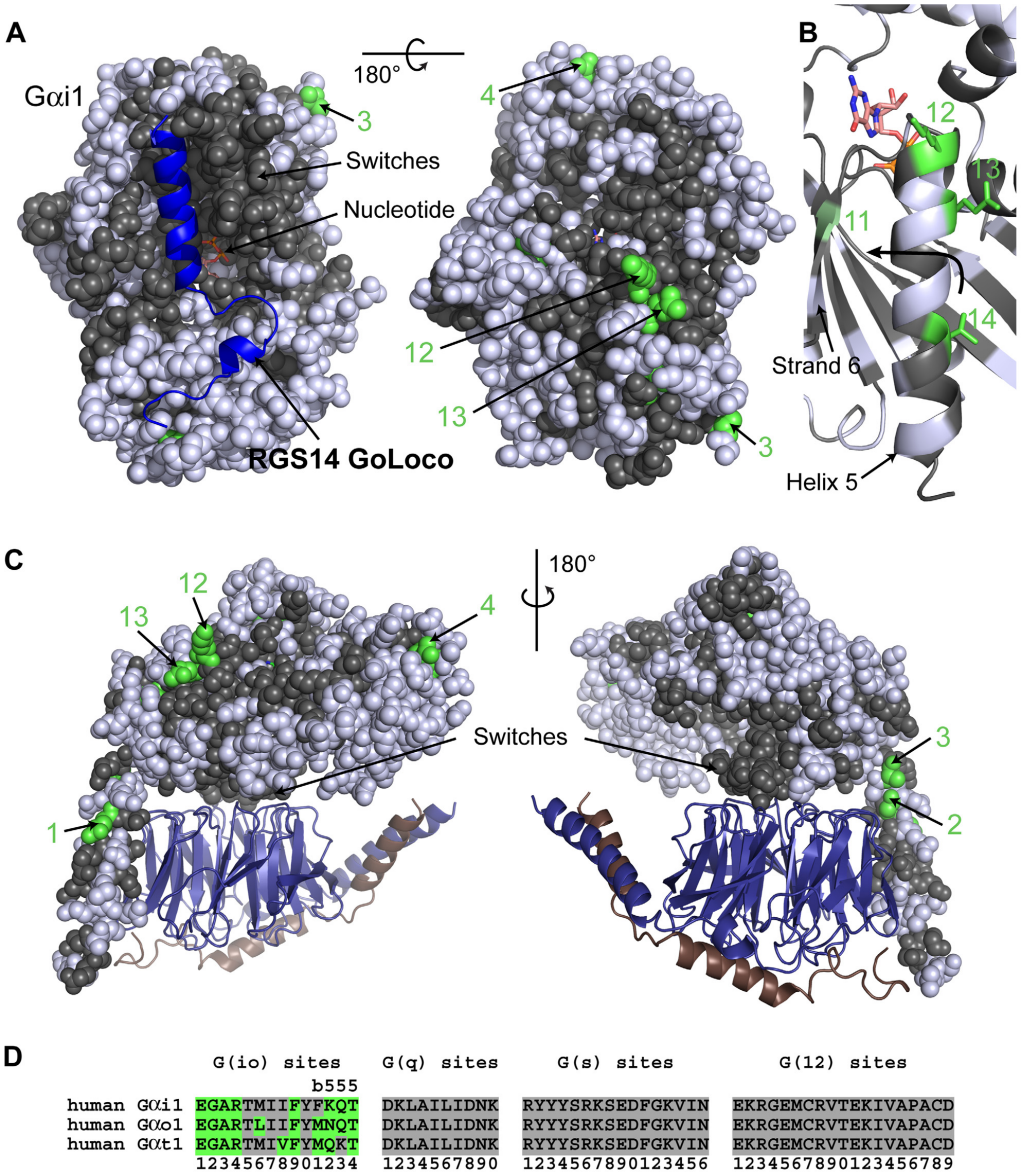
G(io) class-distinctive sites in structural context. (**A**) The structure of the GoLoco domain of RGS14 (blue cartoon) bound to an inactive Gα_i1_ (Gα_i1_ **•** GDP) (PDB ID 1KJY). In all structural panels in this figure, Gα_i1_ is shown as spheres or cartoon with core residues colored gray if they are conserved between Gα subunits (either INV (invariant) or **η** (non-distinct) amino acids) while G(io)-distinctive sites are colored green only if they contain a **∂** (distinct) amino acid. Non-core residues and **d** sites are colored white. G(io)-distinctive sites are numbered according to their position in the signature sequence (see panel (D)). Panel A shows two views of Gα_i1_ related by a 180° rotation about the horizontal axis. The left view is of the switch region of Gα_i1_ while the right view is of the top of the subunit. (**B**) Closeup view of β-strand 6 and α-helix 5 from Gα_i1_ with the side chains of G(io)-distinctive residues in a stick rendering. The orientation is the same as the right-hand view in panel A. Helix 5 rotates and translates toward β-strand 6 (arrow) during GPCR-mediated activation of the Gα subunit. Sites 12 and 13 in Gα_i1_ are the **∂** residue but show subtype variation within the G(io) class. G(io) site 11 (colored lime green) lies in β-strand 6 and also shows subtype variation; Gα_i1_ possesses the **η** residue at that site and is, therefore, colored gray in (A). (**C**) Structure of Gα_i1_ **•**GDP (sphere display) bound to Gβ**•**Gγ (deepblue/copper cartoon) heterodimer (PDB ID 1GP2). Two orientations related by a 180° rotation about the vertical axis are shown. (**D**) Signature sequences are formed by grouping all distinctive sites for a given class together, removing all residues between individual distinctive sites of the noted class. The distinctive sites for each class are presented in order from the N-terminus to the C-terminus and numbered accordingly. Amino acids that correspond to the **∂** values at the G(io) site are colored green. Sites in helix 5 that have been proposed to be important for coupling to the GPCR are denoted by ‘5’ above the site, while site 11 in strand 6 is denoted by ‘b’ above the site.

Though there are two **d** sites in switch III of G(io) family members, there are no G(io)-distinctive sites in the switches of all three subtypes (Gα_i_, Gα_o_, Gα_t_) of the G(io) family members (Figure 7A, left view); all of the G(io)-distinctive sites lie on the opposite face of the molecule (Figure 7A, right view) or are buried. The lack of G(io)-distinctive sites on the switch side of the molecule implies that this family of Gα subunits has maintained the parental functionality in all switches and, therefore, continues to interact with the primordial set of effectors and regulators. While the interface remained ancestral, new effectors – such as GoLoco motifs [42] (Figure 7A, left view) or PDEγ [18] – that utilized surface areas of the parental structure emerged in metaozoans.

Most of the G(io)-distinctive sites on the opposite face of the subunit (Figure 7A, right view) tend to lie in regions associated with binding to the GPCR and with GPCR-driven GDP release (7 sites out of 14 total G(io)-distinctive sites) implying modifications to GPCR specificity and Gα nucleotide binding properties. An N-terminal peptide from Gα_t1_ of the G(io) class was reported to competitively inhibit Gα_t1_-rhodopsin interactions [43]. This N-terminal region contains class-distinctive sites from all four classes (Figure 3). A site-specific fluorescence labeling study reported the greatest receptor activation induced intensity changes and emission shifts – indications of a less aqueous accessible environment – at 3 residues within the Gα_i1_ N-terminal helix [44]. Two of these three residues are class-distinctive sites: G(q)-distinctive site 2 and G(12)-distinctive site 3 (Figure 3). Furthermore, another study identified G(s)-distinctive site 1 as being a key determinant of GPCR selectivity in G(q) family subunits [45]. In yet another study that further refined the GPCR contact surface on Gα subunits, we find the first three G(io)-distinctive residues (sites 1, 2 and 3) lie within the 10-amino acid region in the N-terminal helix identified by covalent cross-linking as a site of contact on Gα_t1_ by the GPCR rhodopsin [46] (Figure 3). We hypothesize these class-distinctive sites are key determinants in Gα-GPCR coupling, although subtle and cooperative interactions are also involved [47].

Similarly, previous studies found key sites of GPCR interaction on the C-terminal region of Gα subunits [48]–[52], a region with several class-distinctive sites. However, several of the sites important for GPCR specificity in the C-terminus rapidly evolved and are thus unique to each Gα subtype.

In contrast to Gα-GPCR coupling in which class specificity was conferred by changing intermolecular interfaces, we hypothesize Gα_i_ evolved class specific functionality by changing an intramolecular interface. Three G(io)-distinctive sites within a helix reported to undergo conformational shifts during activation are likely responsible for mediating that conformational shift during the GPCR driven release of GDP in a class specific manner (Figure 7B). Oldham et al. [21] proposed, based on their measured changes in mobility for residues within helix 5, that helix 5 rotates and translates during GPCR induced activation and in conjunction with the release of GDP. All three of the G(io)-distinctive sites in helix 5 were mutated [21]; mutations at G(io)-distinctive sites 12, 13 and 14 decreased rate of receptor-catalyzed exchange, especially site 14, while the mutation at site 13 also affected the basal exchange rate. In a different study, Kapoor et al. [53] reported that mutation V332A in helix 5 of Gα_i1_ increased basal exchange rates. This residue corresponds to **d** site 15 that lies between G(io)-distinctive sites 12 and 13. An additional G(io) site, site 11, that lies in β-strand 6 (Figure 7B) shows subtype variation within the G(io) class, although no experimental evidence yet links modifications at this site to class-specific functionality. Intriguingly, G(io) sites 12 and 13 also show subtype variation within the G(io) class, in stark contrast to the conservation evident at these same sites in the other three classes. These results are consistent with our proposal that the G(io) class of Gα subunits evolved unique properties for this conformational shift.

### Contrasting how different effectors within a class achieved functional diversification: Modifications to ancestral functionality drive the Gα_q_•GRK2 interaction but not the Gα_q_•p63RhoGEF interaction

We proposed earlier that G(q)-distinctive sites evolved to drive specificity of G(q) interactions to GRK2 (Figure 4A) but not to p63RhoGEF (Figure 4B). In contrast to the Gα_i/q_ **•**GRK2 interface in which 80% of the Gα residues within 4 Å of GRK2 are either invariant or G(q)-distinctive sites, only 50% of the Gα residues at the Gα_i/q_ **•**p63RhoGEF interface have core functionality, either through invariance or G(q)-distinctive sites. This suggests the Gα_q_ **•**p63RhoGEF interaction arose through *de novo* evolution of neo-functionality with the acquired utilization of residues that were not previously functional, rather than primarily through modifications to parental functionality like the Gα_q_ **•**GRK2 interaction. This hypothesis is further supported by noting that the Gα_q_ interface with p63RhoGEF, containing 30 residues, is twice the size of the Gα_q_ **•**GRK2 interface, which contains only 15 residues. Therefore, evolving the Gα_q_ **•**p63RhoGEF interface required fixing an additional 15 residues beyond those used in parental functionality, enough residues to warrant identifying this as *de novo* evolution of an interaction interface.

## Discussion

Gα subunits of G proteins are essential for signal transduction in all eukaryotes. As eukaryotes diversified and became more functionally complex, so did Gα subunits. Extant Gα subunits arose through multiple rounds of duplication and divergence [54]. How these gene duplicates functionally diversified, however, is not well understood. Because Gα resides at the nexus of many signaling pathways and interacts with many effectors, any change can have profound negative pleiotropic effects. How then do highly constrained proteins like Gα evolve the functional complexity we see today? We hypothesize that a narrow subset of class-distinctive sites has the evolutionary potential to confer class-distinctive function with minimal evolutionary cost. From a structural point of view, these sites are those that can mutate and shift the class functionality with a minimal deleterious effect on other aspects of the signaling nexus. At first blush, this idea that highly constrained sites are the ones that confer class specificity is counter-intuitive. Part of the explanation is simple; we found class specificity in the core because we seeded this analysis with the conserved sites within and between classes and avoided highly-labile sites because lack of conservation provides little information about the molecular evolution. Another reason we focused on conserved residues is because changes in these residues have known functional consequences, thereby making any observed class-distinctive change in these sites likely critical for class specific function. We identified 59 of these sites spread across the 14 mammalian Gα. In several instances, these class-distinctive sites associate with known class-specific properties. We also identified many more uncharacterized sites that likely play a role in the sub-functionalization of mammalian Gα. While we have probably not identified all of the residues important for class-distinctive behavior, we identified an important subset of these residues. Mutations at our selected sites will likely disrupt a class-distinctive functionality, but are not likely to be sufficient to confer a full gain of functionality. Other residues, both neutral and restrictive [10], [11], most likely occurred but would not have been identified by our approach.

Our analyses suggested several interesting evolutionary patterns. We showed how two changes at G(q)-distinctive sites determine the specificity of the GRK2 interaction with Gα_q_ and how changes at G(q)-distinctive sites are effector specific, driving specificity of the Gα_q_ **•**GRK2 interaction but not the Gα_q_ **•**p63RhoGEF interaction. We also highlighted the role of G(12)-distinctive sites in the specificity of the Gα_13_ **•**p115RhoGEF interactions. Two of these three examples illustrates how functional diversity within and between classes was driven by changes to parental functionality at class-distinctive sites, while the third example, p63RhoGEF, showed emergence of new functionality by utilizing previously non-constrained residues. In this case, it is possible that the interface evolved in two stages – originally the extended PH domain of p63RhoGEF could have bound to the parental structure of the switch region and the DH domain could have evolved contacts over time to a non-parental surface area (Figure 4B). This process would be mechanistically similar to that speculated for the phosducin interaction with the Gβ subunit [7]. We also showed how evolution can overcome the complexity of G protein interactions by producing structurally-related Gα subunits with opposing functional outcomes. For instance, we showed that by evolving class-distinctive sites that induced conformational changes in Gα, Gα proteins shift from inhibiting AC to stimulating AC. All of these changes affected only the active/transition states of Gα while leaving the inactive state intact and able to interact with the heterodimeric Gβγ complex (Figures 4C, 5C, 6C and 7C). Although two distinctive residues, G(12)-D site 10 and G(s)-D site 8 lie at the interface with the Gβγ complex and have the potential to confer specificity to the interaction of Gα with Gβγ, we are not aware of any published data suggesting there is specificity in this interaction. Finally, we illustrated how novel functionality evolves by variations at sites involved in functionally important conformational changes related to Gα activation rather than through evolution of new interfaces.

**Figure 8:**
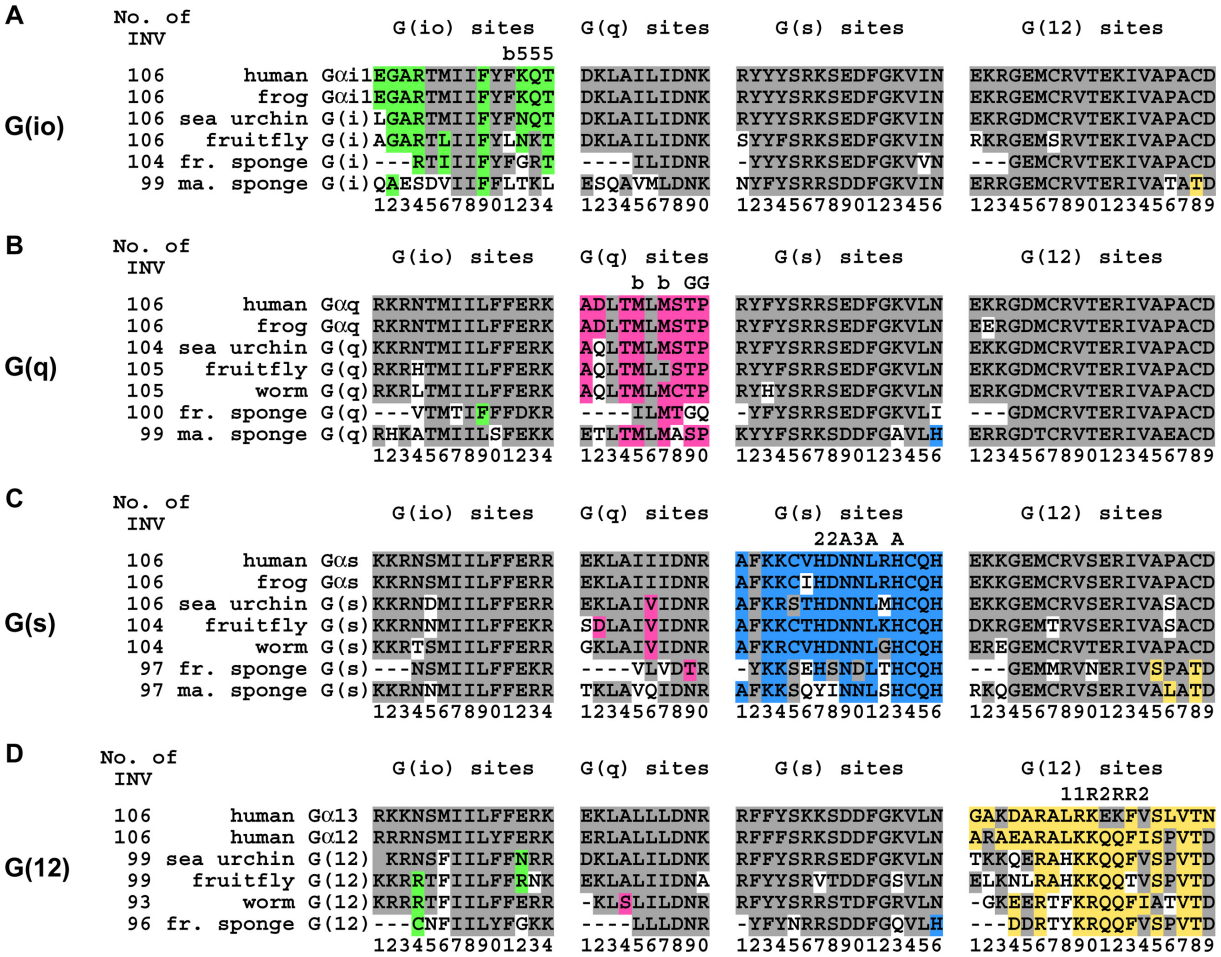
Class-distinctive signature sequences of Gα family members from select organisms. Signature sequences from select organisms are used to follow the evolution of class-distinctive sites (also see Figure 2). Each panel reflects the evolutionary history of a subtype: (**A**) Gα_i1_, (**B**) Gα_q_, (**C**) Gα_s_, (**D**) both Gα_12_ and Gα_13_. Signature sequences are formed by grouping all distinctive sites for a given class together, removing all residues between individual distinctive sites of the noted class. The distinctive sites for each class are presented in order from the N-terminus to the C-terminus and numbered accordingly. Amino acids that correspond to the **∂** values at that site are colored according to distinctive class: green = G(io); magenta = G(q); blue = G(s); and yellow orange = G(12). Class-distinctive sites were determined using only mammalian sequences. Occasionally a non-mammalian subunit will contain a **∂** or variable (white) amino acid where only **η** amino acids were observed in the mammalian sequences (for comparison see Figures 4D, 5D, 6D, 7D). (**A**) Class-distinctive signature sequences of G(io) family members from select organisms. Sites in helix 5 that have been proposed to be important for coupling to the GPCR are denoted by ‘5’ above the site, while site 11 in strand 6 is denoted by ‘b’ above the site. (**B**) Class-distinctive signature sequences of G(q) family members from select organisms. Sites that interact with GRK2 are denoted by ‘G’ above the site, while sites that are buried and not visible are denoted by ‘b’ above the site. (**C**) Class-distinctive signature sequences of G(s) family members from select organisms. Sites that have been proposed to be important for the interaction with adenylyl cyclase are denoted by ‘A’ above the site, while additional sites in switches II or III are denoted by ‘2’ or ‘3’, respectively, above the site. (**D**) Class-distinctive signature sequences of G(12) family members from select organisms. Sites that have direct interactions with p115RhoGEF are denoted by ‘R’ above the site, while additional sites in switches I or II are denoted by ‘1’ or ‘2’, respectively, above the site. Marine sponge = *Geodia cydonium*; Freshwater sponge = *Ephydatia fluviatilis*; Worm = *Caenorhabditis elegans*; Fruitfly = *Drosophila melanogaster*; Sea urchin = *Strongylocentrotus purpuratus*; Frog = *Xenopus laevis*; Human = *Homo sapiens*.

All four Gα classes were formed early in metazoan evolution. From the number of distinctive sites established in the lowest metazoans and the correlations of these changes with class-specific function, our data suggest that the four major Gα classes were established by the split with sponges, in agreement with two earlier studies of Gα evolution [55], [56]. Gα evolution is characterized by bursts of duplication and diversification followed by long quiescent periods [55] and this is also true for class-distinctive sites (Figure 8). For example, our data suggest that the evolution of the class-distinctive sites critical for the GRK2 interaction with Gα_q_ (Figure 8B, G(q) sites 9, 10) occurred around the time of emergence of the G(q) class. However, the GRK2 interaction was not the only sub-functionality driving the emergence of G(q) as several other class-distinctive sites not likely involved in the Gα_q_ **•**GRK2 interaction also appeared at the time of emergence (Figure 8B, G(q) sites 4, 5, 7), and other sites clearly became class-distinctive at later times (Figure 8B, site 1). For G(s) subunits, the G(s) sites associated with AC functionality were also established at the time of emergence of the G(s) class (Figure 8C, G(s) sites 9, 11, 13). At the same time, we see **∂** amino acids became fixed at G(s) sites 1, 15, and 16, sites which are structurally adjacent (Figure 6C, right view). This set of sites is in the GPCR coupling region. We speculate that the evolution of new GPCR specificity was linked to AC activation, resulting in a new signaling network. G(s) site 14 in helix 5, the helix associated with GPCR induced activation, also became distinctive with emergence of the G(s) class, potentially imparting new exchange properties to this signaling pathway. Two additional G(s) sites that currently are not correlated with any known function – G(s) sites 3 and 4 – were also established early, whereas several G(s) sites became distinctive later in evolution. We see similar patterns within the G(12) class.

Interestingly, G(io) sites 12, 13, and 14 – the 3 G(io) sites in helix 5 discussed above – show variance in early metazoans in G(io) and G(12) subunits but not in G(q) or G(s) subunits (Figure 8). This implies an exploration of nucleotide-binding properties in early metazoan ancestors or lineage-specific modifications in G(io) and G(12), but not the other two classes of Gα subunits. Lastly, we see that two G(12)-distinctive sites in switch II (sites 11, 12) possessed **∂** amino acids in the single invertebrate G(12) family member throughout early metazoan evolution but reverted to **η** amino acid values in Gα_13_ after a gene duplication that occurred with the emergence of vertebrates (Figure 8D). This accepted change went from **∂** to **η** – an unusual direction – rather than the canonical direction of **η** to **∂**.

Our data show that the four Gα classes acquired class-distinctive sites throughout metazoan evolution, usually along with the evolution of an expanded or novel class-specific function. One particularly significant period for distinctive site acquisition was before nematodes split from the mammalian lineage (Figure 8) a time when the olfactory system greatly evolved. Interestingly, the second most significant period occurred during the emergence of vertebrates, when all four Gα classes experienced gene duplications leading to an explosion of Gα subtypes, a period when endocrine system complexity dramatically increased. Both olfaction and hormone signaling rely on G protein coupled signaling. This observation may mean that Gα diversification played a critical role in the morphological and physiological evolution of the modern vertebrate.

We propose that specific sequence changes that occurred early in the acquisition of class-specific functionality arose from modifications to parental functionality. Most class-distinctive sites were in regions that were already constrained by functional demands – such as the switches – where modifications to surprisingly few residues could complement existing functionality while simultaneously contributing to divergence. As suggested by Conant and Wolfe [57], the “new” function may have been a secondary property that was always present in the ancestor, similar to the property recently revealed for steroid hormone receptors [11]. Our observation that many of the class-distinctive sites arose from highly conserved residues and essential structural components suggests that gene duplication was essential for the diversification of the Gα. Reduction of the functional constraint on a new paralog following duplication allowed that copy of Gα to convert its secondary property into its primary. This is not, however, the sole mechanism for evolutionary divergence. Both the lack of sequence conservation in the Gα_q_ **•**p63RhoGEF interface and the presence of residues with different evolutionary rates in the α-helical domain [15] imply divergence by evolution of neo-functionality of a previously unspecialized but highly constrained domain following gene duplication.

We believe that our comprehensive view of Gα evolution shows us the amino acid changes that allowed G proteins – despite the constraints put upon them by their myriad of interactions – to become functionally diversified proteins. With this view, we explained several conundrums regarding the structure and function of specific Gα. We produced a partial list (Table S2 and Table S3) of the sites that are likely to contribute to class specific function, and are important for the role of Gα as a signaling nexus. Translating the patterns of evolution at Gα class-distinctive sites into predictions for future structural and functional studies is the next challenge. We will achieve this by uncovering additional details in metazoans of class divergence and the acquisition of neo-functionality in Gα and also by defining the characteristics of the primordial Gα through analysis of the pre-metazoan plant and fungal Gα.

## Materials and Methods

### Gα Sequence Inventory

G-protein sequences were collected from the UniProtKB/Swiss-Prot/TrEMBL Knowledgebase [58] available on the ExPASy Proteomics Server (www.expasy.org) [59] using BLAST [60] and filtered for redundancy using the Ensembl Genome Browser (www.ensembl.org) [61]. Sequences were aligned using ClustalX [62] and adjusted using a T-Coffee alignment program [63] and finally by eye using known atomic structure data as a guide. The final multiple sequence alignment (MSA) contained 347 sequences. Four Gα classes and 16 subclasses were tabulated (Table S1). A subset of sequences was selected for distinctive site determination based on the following criteria: 1) must be mammalian, 2) must be at least human and rodent sequences available for every subclass included in the analysis (Gα_t3_ had only a rat sequence at the time of the original analysis), and 3) subtypes must not be highly divergent (e.g. this excluded Gα_z_ and Gα_15_). Ultimately, a total set of 58 mammalian sequences from all 4 major classes comprising 14 subclasses were culled from the full MSA and used for the final analysis.

Gα sequences from several lower metazoans were included in the analysis reported here. *Geodia cydonium*, a marine sponge, and *Ephydatia fluviatilis*, a freshwater sponge, belong to the phylum Porifera. *G. cydonium* has three Gα proteins: a G(io), a G(q) and a G(s), while *E. fluviatilis* has five proteins that are clear progenitors of mammalian proteins. *E. fluviatilis* has a single G(q), G(s) and G(12) family member, but two G(io)-like members (a G(i) and a G(o)). There are four additional Gα proteins specific to the *Ephydatia* lineage and these were not included in our analysis here. All *E. fluviatilis* sequences used in this study are fragments missing the first ∼50 residues of the amino terminus.

The Gα family *Caenorhabditis elegans* (nematode) expanded greatly, with 21 Gα proteins in total, but only four subunits – G(o), G(q), G(s) and G(12) – are clearly related to the progenitors of mammalian proteins and are thus included in this analysis. The fruit fly, *D. melanogaster* belonging to the phylum Arthropoda, also has G(i), G(o), G(q), G(s), and G(12) members with three additional Gα subunits that are specific to the insect lineage. *Strongylocentrotus purpuratus* (purple sea urchin) is an echinoderm and is the last invertebrate considered in this analysis. Four *S. purpuratus* Gα sequences were analyzed: a G(i), G(q), G(s) and G(12). A number of gene duplications occurred between invertebrates and vertebrates and, given the current available sequences in the databases, it appears that most vertebrates possess a full complement of the 16 mammalian Gα subunits with some having taxa-specific subunits. *Xenopus laevis* (frog) was the model vertebrate organism included in our analysis. Four *X. laevis* Gα sequences were analyzed, annotated as Gα_i1_, Gα_o1_, Gα_q_ and Gα_s_. Current data suggest that the ancestral plant had a single Gα subunit while most extant fungi have two or three Gα proteins. An annotated version of the MSA containing the 58 mammalian sequences used for determining the class-distinctive residues highlighted in Figure 3 and the metazoan sequences included in the evolutionary analysis in Figure 8 is provided in FASTA format (Text S2).

### Mutual Information

Mutual information can be used to measure the correlation between amino acid value and protein family for a set of sequences subdivided into families with different functional specificity (Basharin, 1959). Positions in the alignment which exhibit conservation within each family and variation between families have high mutual information. Positions that exhibit conservation between families (such as invariant residues) or variation within families (such as non-conserved residues) have low mutual information. This method was used by [64] to detect putative specificity-determining residues for paralogous protein kinases. In their study, mutual information was defined as
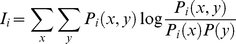
Where *i* is the position in the alignment, *x* the amino acid value, and *y* the protein family number. The summations are over all families in the alignment (*y*) and amino acid values (*x*). *P_i_* (*x*, *y*) is the probability of finding amino acid value *x* at position *i* and in family *y*; *P_i_* (*x*) is the probability of finding amino acid value *x* at position *i* regardless of family; and *P*(*y*) is the fraction of proteins belonging to family *y*. In our mutual information calculation, we subdivided our sequences into four families: G(io), G(q), G(s), and G(12). We also treated amino acid residues with similar side chains as identical, resulting in an amino acid alphabet of 15 values (G, A, V, I = L, M, P, F = Y, W, S = T, N, Q, C, K = R, H, D = E). In addition, we normalized the mutual information scores to the range [0.0,1.0].

### Invariant and Class Distinctive Positions

Sites of interest were characterized as either invariant or class-distinctive. Invariant sites contained the identical amino acid values for the 58 mammalian Gα subunits from all four of the major animal classes. These residues likely were constrained early in Gα evolution and formed part of the primordial Gα core. While invariant sites are important for our understanding of the structural/functional aspects of Gα subunits, they do not contribute to an understanding of the evolution of Gα classes. Each class-distinctive site is occupied by an invariant amino acid (designated **η** – not distinct) in all sequences except for those of a specific functional or distinctive class. Within the distinctive class, sequences contain a different amino acid value (designated **∂** – distinct). In 33 of our 59 sites, the distinctive amino acid **∂** is conserved across all subtypes within the class. Of our 59 sites, 21 show subtype variation within the class. In 5 of our 59 sites, the **∂** amino acid was not conserved for a single subtype and the variation potentially occurred in a non-human sequence.

A single mutual information calculation simultaneously using all four Gα classes cannot identify our selective distinctive sites. Therefore, we used a series of six pair-wise mutual information calculations covering all possible pairs of Gα classes [G(io) vs. G(q); G(io) vs. G(s); G(io) vs. G(12); G(q) vs. G(s); G(q) vs. G(12); G(s) vs. G(12)], then scanned for patterns in the scores to identify distinctive sites (see Table S4). Invariant sites corresponded to positions with the lowest *I_i_* (0.0) for all 6 calculations. Distinctive sites corresponded to positions with the lowest *I_i_* (0.0) for all Gα pairs not involving the distinctive class and higher *I_i_* (>0.0) for all Gα pairs involving the distinctive class.

By accepting any nonzero *I_i_* in the calculations involving determination of distinctive sites, residue positions with a wide range of properties were only tolerated in the distinctive class. The sites had scores that ran from low *I_i_* (0<*I_i_*≪1) for all Gα pairs involving the distinctive class where the position was almost invariant with many **η** and few **∂** values, to residues with high *I_i_* (0<*I_i_*≤1) for all Gα pairs involving the distinctive class and containing only **∂** amino acid values in the distinctive class.

The stringency of criteria for designation as class-distinctive is a function of the amino acids permitted to evolve at that site. Allowing unrestricted evolution, that is any site can evolve to any of the 20 amino acids, would yield only 30 class-distinctive sites instead of the 59 sites identified using a reduced, and more evolutionarily plausible set (see Table S2). Although some substitutions within our reduced amino acid set could result in unaccounted functional changes (e.g. incorporation of a tyrosine phosphorylation site), some sites with known class-distinctive functionality discussed would not have been identified with a 20 amino acid set. We also included class distinctive sites that were identified using an evolutionarily likely set of possible amino acids from our phylogenetic and structural analyses (discussed in the RESULTS section).

### Evolutionary Trace Analysis

We used the Evolutionary Trace Server (ETS) at http://mordred.bioc.cam.ac.uk/~jiye/evoltrace/evoltrace.html to identify evolutionarily important sites for comparison to our class-distinctive sites [65]. We used the identical Gα MSA as utilized for identification of class-distinctive sites, along with chain A of PDB ID 1GP2 for the mapping [17]. Using 10 evenly spaced partitions of our phylogenetic tree, we computed the trace using the TraceSeq and TraceScript algorithms as implemented on the ETS. This revealed the functional patches on the surface of these highly related proteins that reside in similar regions of Gα, regardless of functional differences.

### Modeling of Gα complexes not available in PDB

The homology models of Gα_q_ **•**GDP, Gα_12_ **•**GDP and Gα_s_ **•**GDP each used two partial structures as templates. The first template was an active conformation structure for the given class with the switch regions removed. Structures used as templates – after the removal of the switch regions – were (PDB ID) 2BCJ (Gα_q_), 1AZS (Gα_s_), and 1ZCA (Gα_12_). The switch regions in the three inactive state homology models were built using the switch regions from the inactive Gα_i1_ **•**GDP (PDB ID 1GP2) as the template. Models were generated using InsightII (www.accelrys.com). Side chain rotamer conformations were selected that minimized steric hindrance upon complex formation with the Gβγ subunits from 1GP2. The model of the Gα_12/i1_ **•**p115RhoGEF complex was based on structures of Gα_12/i1_ **•**GDP**•**Mg^2+^ **•**AlF_4_^−^ (PDB ID 1ZCA) and Gα_13/i1_ **•**p115RhoGEF complex (PDB ID 1SHZ). Gα_12_ from 1ZCA was used directly for complex formation with p115RhoGEF except for the adjustment of one side chain conformation to reflect the conformation evident in the complex structure of Gα_13/i1_ **•**p115RhoGEF.

## Supporting Information

Figure S1 Class-distinctive sites in structural context. Gαi1 is in complex with the Gβ•Gγ (deepblue/copper cartoon) heterodimer (PDB ID 1GP2). Gα is shown as spheres (A) or cartoon (B) with core residues colored gray if the residues are conserved between Gα subunits of different classes. All distinctive sites are colored according to the distinctive class (G(io) = green; G(q) = hot pink; G(s) = marine; G(12) = yellow orange). Non-core residues and d sites are colored white. Class-distinctive sites are numbered according to their position in the signature sequence (see Figures 4D, 5D, 6D, 7D, 8). Sites are placed on Gαi1 for relative positioning, no actual mammalian Gα subunit has distinctive sites from more than one class unless it is a chimera.
(2.60 MB PDF)

Table S1 Human Gα classes, subclasses and isoforms.
(0.06 MB PDF)

Table S2 Class-distinctive sites summary.
(0.16 MB PDF)

Table S3 d sites summary.
(0.07 MB PDF)

Table S4 Mutual information calculations.
(0.02 MB PDF)

Text S1 Expanded figure legend for Figure 1, including additional references for noted interactions.
(0.08 MB PDF)

Text S2 Multiple sequence alignment in fasta format.
(0.05 MB TXT)

Text S3 Comparison of evolutionary trace and class-distinctive site analyses.
(0.09 MB PDF)

Text S4 Additional discussion of RhoGEF and G(12) families.
(0.08 MB PDF)

## References

[1] Downes GB, Gautam N (1999). The G protein subunit gene families.. Genomics.

[2] Xu X, Croy JT, Zeng W, Zhao L, Davignon I (1998). Promiscuous coupling of receptors to Gq class α subunits and effector proteins in pancreatic and submandibular gland cells.. J Biol Chem.

[3] Herrmann R, Heck M, Henklein P, Hofmann KP, Ernst OP (2006). Signal transfer from GPCRs to G proteins: role of the Gα N-terminal region in rhodopsin-transducin coupling.. J Biol Chem.

[4] Herrmann R, Heck M, Henklein P, Kleuss C, Wray V (2006). Rhodopsin-transducin coupling: role of the Gα C-terminus in nucleotide exchange catalysis.. Vision Res.

[5] Conant GC, Wolfe KH (2008). Turning a hobby into a job: how duplicated genes find new functions.. Nat Rev Genet.

[6] Jones AM, Assmann SM (2004). Plants: the latest model system for G-protein research.. EMBO Rep.

[7] Friedman EJ, Temple BRS, Hicks SN, Sondek J, Jones CD (2009). Prediction of protein-protein interfaces on G-protein β subunits reveals a novel phospholipase C β2 binding domain.. J Mol Biol.

[8] Kesner BA, Milgram SL, Temple BRS, Dokholyan NV (2009). Isoform divergence of the filamin family of proteins.. Mol Biol Evol.

[9] Bridgham JT, Carroll SM, Thornton JW (2006). Evolution of hormone-receptor complexity by molecular exploitation.. Science.

[10] Bridgham JT, Ortlund EA, Thornton JW (2009). An epistatic ratchet constrains the direction of glucocorticoid receptor evolution.. Nature.

[11] Ortlund EA, Bridgham JT, Redinbo MR, Thornton JW (2007). Crystal structure of an ancient protein: Evolution by conformational epistasis.. Science.

[12] Basharin G (1959). On a statistical estimate for the entropy of a sequence of independent random variables.. Theory Prob Appl.

[13] Martin LC, Gloor GB, Dunn SD, Wahl LM (2005). Using information theory to search for co-evolving residues in proteins.. Bioinformatics.

[14] Lichtarge O, Bourne HR, Cohen FE (1996). Evolutionarily conserved Gαβγ binding surfaces support a model of the G protein-receptor complex.. Proc Natl Acad Sci U S A.

[15] Zheng Y, Xu D, Gu X (2007). Functional divergence after gene duplication and sequence-structure relationship: a case study of G-protein alpha subunits.. Mol Dev Evol.

[16] Lambright DG, Sondek J, Bohm A, Skiba NP, Hamm HE (1996). The 2.0 Å crystal structure of a heterotrimeric G Protein.. Nature.

[17] Wall MA, Coleman DE, Lee E, Iniguez-Lluhi JA, Posner BA (1995). The structure of the G protein heterotrimer Gi_α1β1γ1_.. Cell.

[18] Slep KC, Kercher MA, He W, Cowan CW, Wensel TG (2001). Structural determinants for regulation of phosphodiesterase by a G protein at 2.0 Å.. Nature.

[19] Tesmer JJ, Berman DM, Gilman AG, Sprang SR (1997). Structure of RGS4 bound to AlF_4_
^−^-activated G_iα1_: stabilization of the transition state for GTP hydrolysis.. Cell.

[20] Chen Z, Singer WD, Sternweis PC, Sprang SR (2005). Structure of the p115RhoGEF rgRGS domain-Gα13/i1 chimera complex suggests convergent evolution of a GTPase activator.. Nat Struct Mol Biol.

[21] Oldham WM, Van Eps N, Preininger AM, Hubbell WL, Hamm HE (2006). Mechanism of the receptor-catalyzed activation of heterotrimeric G proteins.. Nat Struct Mol Biol.

[22] Fields TA, Casey PJ (1997). Signalling functions and biochemical properties of pertussis toxin-resistant G-proteins.. Biochem J.

[23] Pitcher JA, Freedman NJ, Lefkowitz RJ (1998). G Protein-coupled receptor kinases.. Annu Rev Biochem.

[24] Koch WJ, Hawes BE, Inglese J, Luttrell LM, Lefkowitz RJ (1994). Cellular expression of the carboxyl terminus of a G protein-coupled receptor kinase attenuates Gβγ-mediated signaling.. J Biol Chem.

[25] Day PW, Carman CV, Sterne-Marr R, Benovic JL, Wedegaertner PB (2003). Differential interaction of GRK2 with members of the Gαq family.. Biochemistry.

[26] Tesmer VM, Kawano T, Shankaranarayanan A, Kozasa T, Tesmer JJG (2005). Snapshot of activated G proteins at the membrane: The Gαq-GRK2-Gβγ complex.. Science.

[27] Lutz S, Freichel-Blomquist A, Yang Y, Rumenapp U, Jakobs KH (2005). The guanine nucleotide exchange factor p63RhoGEF, a specific link between Gq/11-coupled receptor signaling and RhoA.. J Biol Chem.

[28] Rojas RJ, Yohe ME, Gershburg S, Kawano T, Kozasa T (2007). Gαq directly activates p63RhoGEF and trio via a conserved extension of the Dbl homology-associated pleckstrin homology domain.. J Biol Chem.

[29] Souchet M, Portales-Casamar E, Mazurais D, Schmidt S, Leger I (2002). Human p63RhoGEF, a novel RhoA-specific guanine nucleotide exchange factor, is localized in cardiac sarcomere.. J Cell Sci.

[30] Lutz S, Shankaranarayanan A, Coco C, Ridilla M, Nance MR (2007). Structure of Gαq-p63RhoGEF-RhoA complex reveals a pathway for the activation of RhoA by GPCRs.. Science.

[31] Hart M, Jiang J, X., Kozasa T, Roscoe W, Singer WD (1998). Direct stimulation of the guanine nucleotide exchange activity of p115 RhoGEF by Gα13.. Science.

[32] Kozasa T, Jiang X, Hart MJ, Sternweis PM, Singer WD (1998). p115RhoGEF, a GTPase activating protein for G_12_ and G_13_.. Science.

[33] Mao J, Yuan H, Xie W, Wu D (1998). Guanine nucleotide exchange factor GEF115 specifically mediates activation of Rho and serum response factor by the G protein α subunit Gα13.. Proc Natl Acad Sci U S A.

[34] Grabocka E, Wedegaertner PB (2005). Functional consequences of Gα13 mutations that disrupt interaction with p115RhoGEF.. Oncogene.

[35] Nakamura S, Kreutz B, Tanabe S, Suzuki N, Kozasa T (2004). Critical role of lysine 204 in switch I region of Gα13 for regulation of p115RhoGEF and leukemia-associated RhoGEF.. Mol Pharmacol.

[36] Kreutz B, Yau DM, Nance MR, Tanabe S, Tesmer JJ (2006). A new approach to producing functional Gα subunits yields the activated and deactivated structures of Gα_12/13_ proteins.. Biochemistry.

[37] Coleman DE, Berghuis AM, Lee E, Linder ME, Gilman AG (1994). Structures of active conformations of Giα1 and the mechanism of GTP hydrolysis.. Science.

[38] Noel JP, Hamm HE, Sigler PB (1993). The 2.2 Å crystal structure of transducin-α complexed with GTPγS.. Nature.

[39] Sunahara RK, Tesmer JJG, Gilman AG, Sprang SR (1997). Crystal structure of the adenylyl cyclase activator G_sα_.. Science.

[40] Tesmer JJG, Sunahara RK, Gilman AG, Sprang SR (1997). Crystal structure of the catalytic domains of adenylyl cyclase in a complex with G_sα_•GTPγS.. Science.

[41] Berlot CH, Bourne HR (1992). Identification of effector-activating residues of G_sα_.. Cell.

[42] Kimple RJ, Kimple ME, Betts L, Sondek J, Siderovski DP (2002). Structural determinants for GoLoco-induced inhibition of nucleotide release by Gα subunits.. Nature.

[43] Hamm HE, Deretic D, Arendt A, Hargrave PA, Koenig B (1998). Site of G protein binding to rhodopsin mapped with synthetic peptides from the alpha subunit.. Science.

[44] Preininger AM, Parello J, Meier SM, Liao G, Hamm HE (2008). Receptor-mediated changes at the myristoylated amino terminus of Gα_i1_ proteins.. Biochemistry.

[45] Blahos J, Fischer T, Brabet I, Stauffer D, Rovelli G (2001). A novel site on the Gα-protein that recognizes heptahelical receptors.. J Biol Chem.

[46] Itoh Y, Cai K, Khorana HG (2001). Mapping of contact sites in complex formation between light-activated rhodopsin and transducin by covalent crosslinking: Use of a chemically preactivated reagent.. Proc Natl Acad Sci U S A.

[47] Slessareva JE, Ma H, Depree KM, Flood LA, Bae H (2003). Closely related G-protein-coupled receptors use multiple and distinct domains on G-protein α-subunits for selective coupling.. J Biol Chem.

[48] Cai K, Itoh Y, Khorana HG (2001). Mapping of contact sites in complex formation between transducin and light-activated rhodopsin by covalent crosslinking: Use of a photoactivatable reagent.. Proc Natl Acad Sci U S A.

[49] Garcia PD, Onrust R, Bell SM, Sakmar TP, Bourne HR (1995). Transducin-α C-terminal mutations prevent activation by rhodopsin: a new assay using recombinant proteins expressed in cultured cells.. EMBO J.

[50] Natochin M, Granovsky AE, Muradov KG, Artemyev NO (1999). Roles of the transducin α-subunit α4-helix/α4-β6 loop in the receptor and effector interactions.. J Biol Chem.

[51] Onrust R, Herzmark P, Chi P, Garcia PD, Lichtarge O (1997). Receptor and βγ binding sites in the α subunit of the retinal G protein transducin.. Science.

[52] Osawa S, Weiss ER (1995). The effect of carboxyl-terminal mutagenesis of G_t_α on rhodopsin and guanine nucleotide binding.. J Biol Chem.

[53] Kapoor N, Menon ST, Chauhan R, Sachdev P, Sakmar TP (2009). Structural evidence for a sequential release mechanism for activation of heterotrimeric G proteins.. J Mol Biol.

[54] Wilkie TM, Gilbert DJ, Olsen AS, Chen X-N, Amatruda TT (1992). Evolution of the mammalian G protein α subunit multigene family.. Nat Genet.

[55] Suga H, Koyanagi M, Hoshiyama D, Ono K, Iwabe N (1999). Extensive gene duplication in the early evolution of animals before the parazoan-eumetazoan split demonstrated by G proteins and protein tyrosine kinases from sponges and Hydra.. J Mol Evol.

[56] Seack J, Kruse M, Muller WE (1998). Evolutionary analysis of G-proteins in early metazoans: cloning of α-and β- subunits from the sponge, *Geodia cydonium*.. Biochem Biophys Acta.

[57] Conant GC, Wolfe KH (2008). Turning a hobby into a job: How duplicated genes find new functions.. Nat Rev Genet.

[58] Bairoch A, Apweiler R, Wu CH, Barker WC, Boeckmann B (2005). The universal protein resource (UniProt).. Nucl Acids Res.

[59] Gasteiger E, Gattiker A, Hoogland C, Ivanyi I, Appel RD (2003). ExPASy: the proteomics server for in-depth protein knowledge and analysis.. Nucl Acids Res.

[60] Altschul SF, Gish W, Miller W, Myers EW, Lipman DJ (1990). Basic local alignment search tool.. J Mol Biol.

[61] Birney E, Andrews TD, Bevan P, Caccamo M, Chen Y (2004). An overview of ensembl.. Genome Res.

[62] Thompson JD, Gibson TJ, Plewniak F, Jeanmougin F, Higgins DG (1997). The ClustalX windows interface: flexible strategies for multiple sequence alignment aided by quality analysis tools.. Nuc Acids Res.

[63] Notredame C, Higgins DG, Heringa J (2000). T-coffee: a novel method for fast and accurate multiple sequence alignment.. J Mol Biol.

[64] Li L, Shakhnovich EI, Mirny LA (2003). Amino acids determining enzyme-substrate specificity in prokaryotic and eukaryotic protein kinases.. Proc Natl Acad Sci U S A.

[65] Innis CA, Shi J, Blundell TL (2000). Evolutionary trace analysis of TGF-β and related growth factors: implications for site-directed mutagenesis.. Protein Eng.

