# Supplementary material for: Evolution of a Signaling Nexus Constrained by Protein Interfaces and Conformational States
Source: PLoS Comput Biol. 2010 Oct 14;6(10):e1000962. doi: 10.1371/journal.pcbi.1000962 (PMC2954821; doi:10.1371/journal.pcbi.1000962)
Supplement: Figure S1 — Class-distinctive sites in structural context. Gαi1 is in complex with the Gβ•Gγ (deepblue/copper cartoon) heterodimer (PDB ID 1GP2). Gα is shown as spheres (A) or cartoon (B) with core residues colored gray if the residues are conserved between Gα subunits of different classes. All distinctive sites are colored according to the distinctive class (G(io) = green; G(q) = hot pink; G(s) = marine; G(12) = yellow orange). Non-core residues and d sites are colored white. Class-distinctive sites are numbered according to their position in the signature sequence (see Figures 4D, 5D, 6D, 7D, 8). Sites are placed on Gαi1 for relative positioning, no actual mammalian Gα subunit has distinctive sites from more than one class unless it is a chimera. (2.60 MB PDF) [file pcbi.1000962.s001.pdf]

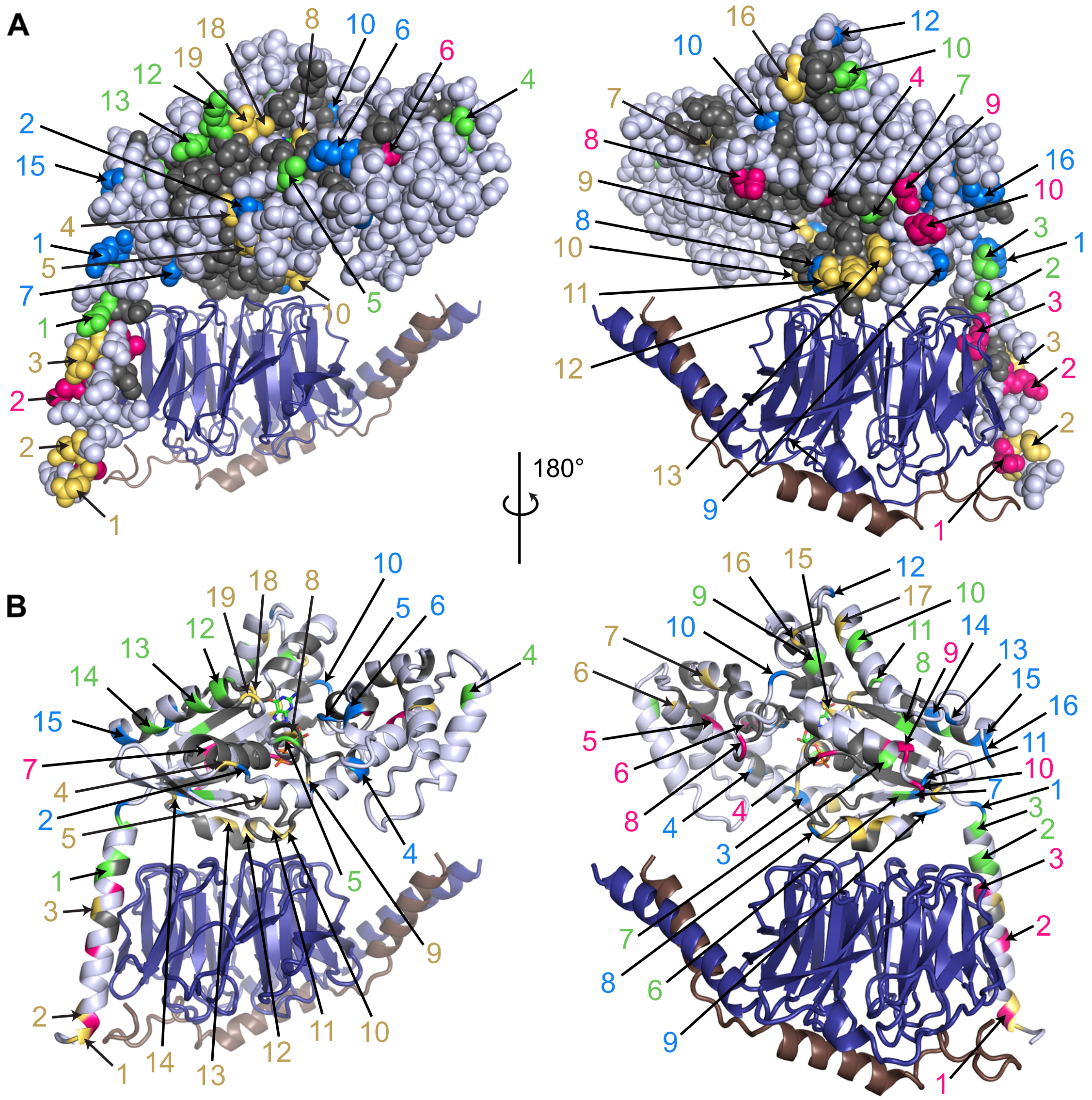

Figure S1
Class-distinctive sites in structural context. Gαi1 is in complex with the Gβ•Gγ (deepblue/copper cartoon) heterodimer (PDB ID 1GP2). Gα is shown as spheres (A) or cartoon (B) with core residues colored gray if the residues are conserved between Gα subunits of different classes. All distinctive sites are colored according to the distinctive class (G(io) = green; G(q) = hot pink; G(s) = marine; G(12) = yellow orange). Non-core residues and d sites are colored white. Class-distinctive sites are numbered according to their position in the signature sequence (see Figures 4D, 5D, 6D, 7D, 8). Sites are placed on Gαi1 for relative positioning, no actual mammalian Gα subunit has distinctive sites from more than one class unless it is a chimera.
