# Supplementary material for: Evolution of a Signaling Nexus Constrained by Protein Interfaces and Conformational States
Source: PLoS Comput Biol. 2010 Oct 14;6(10):e1000962. doi: 10.1371/journal.pcbi.1000962 (PMC2954821; doi:10.1371/journal.pcbi.1000962)
Supplement: Table S1 — Human Gα classes, subclasses and isoforms. (0.06 MB PDF) [file pcbi.1000962.s002.pdf]

Table S1: Human Gα Classes, Subclasses, and Isoforms

Class	Subclass	Isoform	Gene	UniProt Accession	Included in Analysis
G(io)	G(i)	$G\alpha_{i1}$	GNAI1	P63096	Yes
		$G\alpha_{i2}$	GNAI2	P04899	Yes
		$G\alpha_{i3}$	GNAI3	P08754	Yes
	G(o)	$G\alpha_{o1}$	GNAO1	P09471	Yes
		$G\alpha_{o2}$	GNAO1	P09471-2	Yes
	G(t)	$G\alpha_{t1}$	GNAT1	P11488	Yes
		$G\alpha_{t2}$	GNAT2	P19087	Yes
		$G\alpha_{t3}$	GNAT3	A8MTJ3	No
	G(z)	$G\alpha_{z}$	GNAZ	P19086	No
G(q)		$G\alpha_{q}$	GNAQ	P50148	Yes
		$G\alpha_{11}$	GNA11	P29992	Yes
		$G\alpha_{14}$	GNA14	O95837	Yes
		$G\alpha_{15}$	GNA15	P30679	No
G(s)		$G\alpha_{s}$	GNAS	P63092	Yes
		$G\alpha_{olf}$	GNAL	P38405	Yes
G(12)		$G\alpha_{12}$	GNA12	Q03113	Yes
		$G\alpha_{13}$	GNA13	Q14344	Yes
