# Supplementary material for: Evolution of a Signaling Nexus Constrained by Protein Interfaces and Conformational States
Source: PLoS Comput Biol. 2010 Oct 14;6(10):e1000962. doi: 10.1371/journal.pcbi.1000962 (PMC2954821; doi:10.1371/journal.pcbi.1000962)
Supplement: Table S2 — Class-distinctive sites summary. (0.16 MB PDF) [file pcbi.1000962.s003.pdf]

TABLE S2: Class-Distinctive Sites Summary

Site[1]	η amino acid	∂ amino acid (subclass)	Distinctive Class[2]	Position within Class[3]	Comments
**1**	D/E	A ($G\alpha_{12}$)<br>G ($G\alpha_{13}$)[7]	G(12)	#1	
**2**	D/E	A ($G\alpha_{q}$)<br>V ($G\alpha_{11}$)[7,8]	G(q)	#1	
**3**	K/R	A ($G\alpha_{13}$)[5,8]	G(12)	#2	
**4**	K/R	D ($G\alpha_{q}$)<br>A ($G\alpha_{11}$,$G\alpha_{14}$)[7]	G(q)	#2	Less aqueous environment during GPCR-driven activation
**5**	K/R	A ($G\alpha_{12}$) [5,8]	G(12)	#3	Less aqueous environment during GPCR-driven activation
**6**	L	V (mouse $G\alpha_{q}$, $G\alpha_{14}$)[6,8]	G(q)	#3	
**7**	K/R	E ($G\alpha_{i}$,$G\alpha_{o}$,$G\alpha_{t}$)[4]	G(io)	#1	Potential contact to GPCR
**8**	K/R	G ($G\alpha_{i}$,$G\alpha_{o}$)<br>A ($G\alpha_{t}$)[7]	G(io)	#2	Potential contact to GPCR
**9**	K/R	A ($G\alpha_{i}$,$G\alpha_{o}$,$G\alpha_{t}$)[4]	G(io)	#3	Potential contact to GPCR
**10**	K/R	A ($G\alpha_{s}$,$G\alpha_{olf}$)[4]	G(s)	#1	Key for GPCR selectivity in G(q) subunits
**11**	A	T ($G\alpha_{q}$,$G\alpha_{11}$,$G\alpha_{14}$)[4]	G(q)	#4	
**12**	G	D/E ($G\alpha_{12}$,$G\alpha_{13}$)[4]	G(12)	#4	
**13**	F/Y	S (rat $G\alpha_{olf}$)[6,8]	G(s)	#2	
**14**	D/E	A ($G\alpha_{12}$,$G\alpha_{13}$)[4]	G(12)	#5	
**15**	F/Y	K ($G\alpha_{s}$,$G\alpha_{olf}$)[4]	G(s)	#3	
**16**	F/Y	K/R ($G\alpha_{s}$,$G\alpha_{olf}$)[4]	G(s)	#4	
**17**	I/L	M ($G\alpha_{q}$,$G\alpha_{11}$,$G\alpha_{14}$)[4]<br>V (rat $G\alpha_{11}$)[6]	G(q)	#5	
**18**	M	R ($G\alpha_{12}$,$G\alpha_{13}$)[4]	G(12)	#6	
**19**	N	R ($G\alpha_{i}$,$G\alpha_{o}$,$G\alpha_{t1}$)<br>C ($G\alpha_{t2}$)[7]	G(io)	#4	
**20**	C	A ($G\alpha_{12}$,$G\alpha_{13}$)[4]	G(12)	#7	
**21**	S	C ($G\alpha_{s}$,$G\alpha_{olf}$)[4]	G(s)	#5	
**22**	I/L	V ($G\alpha_{11}$)[5,8]	G(q)	#6	
**23**	K/R	V ($G\alpha_{s}$)<br>S ($G\alpha_{olf}$)[7]	G(s)	#6	
**24**	S/T	N (mouse, bovine $G\alpha_{t2}$)[6,8]	G(io)	#5	
**25**	R	L ($G\alpha_{12}$,$G\alpha_{13}$)[4]	G(12)	#8	
**26**	V	K/R ($G\alpha_{12}$,$G\alpha_{13}$)[4]	G(12)	#9	Tethers Switch I to insert in α-helical domain
**27**	S/T	K ($G\alpha_{12}$,$G\alpha_{13}$)[4]	G(12)	#10	Mutation diminishes P115RhoGEF binding
**28**	K/R	H ($G\alpha_{s}$,$G\alpha_{olf}$)[4]	G(s)	#7	
**29**	M	L ($G\alpha_{o}$)[5,8]	G(io)	#6	
**30**	S	D ($G\alpha_{s}$,$G\alpha_{olf}$)[4]	G(s)	#8	
**31**	E	Q ($G\alpha_{12}$)[5,8]	G(12)	#11	
**32**	K/R	Q ($G\alpha_{12}$)[5,8]	G(12)	#12	Contact to p115RhoGEF
**33**	I	F ($G\alpha_{12}$,$G\alpha_{13}$)[4]	G(12)	#13	Contact to p115RhoGEF
**34**	D/E	N ($G\alpha_{s}$,$G\alpha_{olf}$)[4]	G(s)	#9	Mutant slightly reduces adenylyl cylase binding
**35**	V	I ($G\alpha_{12}$)[5,8]	G(12)	#14	
**36**	I/L	M ($G\alpha_{q}$,$G\alpha_{11}$)[5,8]	G(q)	#7	

**37**	A	S ($G\alpha_{12}$,$G\alpha_{13}$)[4]	G(12)	#15	
**38**	D	N ($G\alpha_{s}$,$G\alpha_{olf}$)[4]	G(s)	#10	
**39**	D	S ($G\alpha_{q}$,$G\alpha_{11}$)<br>C ($G\alpha_{14}$)[7]	G(q)	#8	
**40**	I	T (orangutan $G\alpha_{i1}$)[6,8]	G(io)	#7	
**41**	N	T ($G\alpha_{q}$,$G\alpha_{11}$,$G\alpha_{14}$)[4]	G(q)	#9	Contact to GRK2
**42**	K/R	P ($G\alpha_{q}$,$G\alpha_{11}$,$G\alpha_{14}$)[4]	G(q)	#10	P➔K mutation eliminates GRK2 binding
**43**	F	L ($G\alpha_{s}$,$G\alpha_{olf}$)[4]	G(s)	#11	Important for adenylyl cyclase binding
**44**	I	V ($G\alpha_{t}$)[5,8]	G(io)	#8	
**45**	L	F ($G\alpha_{i}$,$G\alpha_{o}$,$G\alpha_{t}$)[4]	G(io)	#9	
**46**	P	L ($G\alpha_{13}$)[5,8]	G(12)	#16	
**47**	G	R ($G\alpha_{s}$)<br>N ($G\alpha_{olf}$)[7]	G(s)	#12	
**48**	A	V ($G\alpha_{12}$,$G\alpha_{13}$)[4]	G(12)	#17	
**49**	F/Y	H (rodent $G\alpha_{o2}$)[6,8]	G(io)	#10	
**50**	K/R	H ($G\alpha_{s}$,$G\alpha_{olf}$)[4]	G(s)	#13	Important for adenylyl cyclase binding
**51**	F	V ($G\alpha_{o2}$)[6]<br>M ($G\alpha_{o1}$,$G\alpha_{t}$)[7,8]	G(io)	#11	
**52**	C	T ($G\alpha_{12}$,$G\alpha_{13}$)[4]	G(12)	#18	
**53**	D	N ($G\alpha_{13}$)[5,8]	G(12)	#19	
**54**	D/E	K ($G\alpha_{i}$)<br>N ($G\alpha_{o}$)<br>Q ($G\alpha_{t}$)[7]	G(io)	#12	Mobility changes during GPCR-driven activation, decreased rate of receptor-catalyzed exchange
**55**	K/R	Q ($G\alpha_{i}$,$G\alpha_{o}$)[5,8]	G(io)	#13	Mutation increased basal rate of exchange, decreased rate of receptor-catalyzed exchange
**56**	V	C ($G\alpha_{s}$,$G\alpha_{olf}$)[4]	G(s)	#14	
**57**	K/R	T ($G\alpha_{i}$,$G\alpha_{o}$,$G\alpha_{t}$)[4]	G(io)	#14	Mutation decreased rate of receptor-catalyzed exchange
**58**	I/L	Q ($G\alpha_{s}$,$G\alpha_{olf}$)[4]	G(s)	#15	
**59**	N	H ($G\alpha_{s}$,$G\alpha_{olf}$)[4]	G(s)	#16	

[1] Corresponds to the order of sites in Figure 2.

[2] G(io), G(q), G(s), and G(12) sites denoted by 'I', 'Q', 'S', and '2', respectively, in Figure 2.

[3] Corresponds to the site number given in Figures 3-6 and the order of sites in Figure 7.

[4] Single ∂ amino acid in <u>all</u> subclasses within distinctive class

[5] Single ∂ amino acid in some but not all subclasses within distinctive class

[6] Single ∂ amino acid in just a few sequences (but not an entire subclass) within distinctive class

[7] ∂ amino acid varies with subclass within distinctive class

[8] η amino acids found in distinctive class
