# Supplementary material for: Evolution of a Signaling Nexus Constrained by Protein Interfaces and Conformational States
Source: PLoS Comput Biol. 2010 Oct 14;6(10):e1000962. doi: 10.1371/journal.pcbi.1000962 (PMC2954821; doi:10.1371/journal.pcbi.1000962)
Supplement: Table S3 — d sites summary. (0.07 MB PDF) [file pcbi.1000962.s004.pdf]

TABLE S3: d Sites Summary

d Site[1]	η amino acid	∂ amino acid (subclass)	Distinctive Class	∂ amino acid (subclass)	Distinctive Class	Class-Distinctive Site(s) Within 5 Å
**1**	S	N ($G\alpha_s$,$G\alpha_{olf}$)	**G(s)**	N ($G\alpha_q$,$G\alpha_{11}$)	G(q)	**G(q)-D#2**
**2**	D/E	G ($G\alpha_s$)	**G(s)**	K/R ($G\alpha_{12}$,$G\alpha_{13}$)	G(12)	**G(12)-D #5**
**3**	K/R	C ($G\alpha_i$,$G\alpha_t$)<br>V ($G\alpha_o$)	G(i)	L ($G\alpha_{12}$)	G(12)	**G(12)-D #5**
**4**	Q	K/R ($G\alpha_s$,$G\alpha_{olf}$)	**G(s)**	R ($G\alpha_{12}$)	G(12)	**G(12)-D#7**
**5**	D/E	N ($G\alpha_o$,$G\alpha_{i1}$)<br>S ($G\alpha_{i3}$)<br>G ($G\alpha_{i2}$)	G(io)	S ($G\alpha_{12}$)	G(12)	**G(12)-D#7**
**6**	K	A,S ($G\alpha_i$)<br>S,G ($G\alpha_t$)	G(io)	Q ($G\alpha_s$,$G\alpha_{olf}$)	G(s)	**G(s)-D#5**
**7**	D/E	N ($G\alpha_i$,$G\alpha_{t2}$)<br>S ($G\alpha_{t1}$)	G(io)	N ($G\alpha_q$)<br>T ($G\alpha_{11}$,$G\alpha_{14}$)	G(q)	**G(q)-D#6**
**8**	Q	L ($G\alpha_i$)<br>M ($G\alpha_t$)	G(io)	M ($G\alpha_s$,$G\alpha_{olf}$)	G(s)	**G(s)-D#10**
**9**	T	M ($G\alpha_i$)<br>V ($G\alpha_t$)	G(io)	E ($G\alpha_q$,$G\alpha_{11}$,$G\alpha_{14}$)	G(q)	**G(q)-D#8**
**10**	N	H ($G\alpha_t$)	G(io)	Y ($G\alpha_q$,$G\alpha_{11}$,$G\alpha_{14}$)	G(q)	**G(q)-D#9**<br>**G(q)-D#10**<br>**G(io)-D#7**
**11**	W	F/Y ($G\alpha_{o1}$,$G\alpha_t$)	G(io)	L ($G\alpha_{12}$)<br>V ($G\alpha_{13}$)	G(12)	**G(12)-D#13**
**12**	I/L	M ($G\alpha_{olf}$)	**G(s)**	V ($G\alpha_{t1}$)	G(io)	**G(io)-D#9**
**13**	Y	C ($G\alpha_i$,$G\alpha_o$,$G\alpha_t$)	G(io)	H ($G\alpha_{12}$)	G(12)	**G(12)-D#16**
**14**	I/L	V (rodent $G\alpha_{o2}$)	G(io)	C ($G\alpha_s$,$G\alpha_{olf}$)	G(s)	**G(s)-D#13**
**15**	I	V (human $G\alpha_{12}$)	G(12)	V ($G\alpha_i$,$G\alpha_t$)	G(io)	**G(io)-D#12**<br>**G(io)-D#13**<br>**G(12)-D#18**<br>**G(12)-D#19**
**16**	A	D ($G\alpha_{13}$)	G(12)	D ($G\alpha_s$)	G(s)	**G(s)-D#14**

[1] Corresponds to the order of sites in Figure 2.
