# Supplementary material for: Evolution of a Signaling Nexus Constrained by Protein Interfaces and Conformational States
Source: PLoS Comput Biol. 2010 Oct 14;6(10):e1000962. doi: 10.1371/journal.pcbi.1000962 (PMC2954821; doi:10.1371/journal.pcbi.1000962)
Supplement: Table S4 — Mutual information calculations. (0.02 MB PDF) [file pcbi.1000962.s005.pdf]

Table S4: Class-Distinctive Sites Defined by Series of Mutual Information Calculations

		Classes Included in MI Calculation at a Given Site[1]					
		G(io) & G(q)	G(io) & G(s)	G(io) & G(12)	G(q) & G(s)	G(q) & G(12)	G(s) & G(12)
	Invariant[2]	0.0	0.0	0.0	0.0	0.0	0.0
**Class Distinctive Site**	G(io)-D[3]	>**0.0**	>**0.0**	>**0.0**	0.0	0.0	0.0
	G(q)-D[3]	>**0.0**	0.0	0.0	>**0.0**	>**0.0**	0.0
	G(s)-D[3]	0.0	>**0.0**	0.0	>**0.0**	0.0	>**0.0**
	G(12)-D[3]	0.0	0.0	>**0.0**	0.0	>**0.0**	>**0.0**

[1]Six independent MI calculations were carried out, each MI calculation involving sequences from only 2 of the 4 Gα classes.
[2]A site was defined as “Invariant” when the MI score was 0.0 for all 6 MI calculations at that site.
[3]A site was defined as “Distinctive” for a given Gα class when the six MI scores at that site fit the criteria established for that class.
