# Supplementary material for: Evolution of a Signaling Nexus Constrained by Protein Interfaces and Conformational States
Source: PLoS Comput Biol. 2010 Oct 14;6(10):e1000962. doi: 10.1371/journal.pcbi.1000962 (PMC2954821; doi:10.1371/journal.pcbi.1000962)
Supplement: Text S1 — Expanded figure legend for Figure 1, including additional references for noted interactions. (0.08 MB PDF) [file pcbi.1000962.s006.pdf]

FIGURE 1

Gα as a regulated molecular signaling nexus. This graphic of the Gα signaling nexus delineates functional elements within the molecule such as nucleotide binding (e.g. NKxD motif, TCAT motif, P-loop) and GPCR-driven nucleotide exchange (α-helix 5, β-strand 6), the different conformations of Gα (i.e. transition and GDP-, GTP-bound states), along with mammalian macromolecules that have been reported to directly interact with Gα. Reported interactions are classified by nucleotide dependence and by functional outcome (GDI, GEF, etc.). The single-headed arrow represents an interaction leading to signaling, the cross represents an interaction that does not lead to signaling, a blunt arrow represents interactions leading to signal termination, while the double-headed arrow represents a neutral physical interaction. While the list of reported interactions is intended to be extensive, it is not intended to be exhaustive, particularly in regard to the GPCRs.

References for the interactions included in the graphic are given below:

Interactions with GPCRs:

Interactions between GPCRs and Gα family members are discussed in the following references: (Juneja and Casey 2009), (Hubbard and Hepler 2006).

Nucleotide-Free State Interactions:

GIV (Gα-interacting vesicle-associated protein) was shown to directly interact with the GDP-bound state of Gαi and to also act as a nonreceptor GEF for the subunit (Garcia-Marcos, Ghosh, and Farquhar 2009; Ghosh et al. 2008; Le-Niculescu et al. 2005). Ric-8A interacted

preferentially with the GDP-bound Gα subunits and catalyzed exchange of GDP for GTP *in vitro* by stabilizing the nucleotide-free conformation (Tall, Krumins, and Gilman 2003; Tall and Gilman 2004; Tall and Gilman 2005; Thomas et al. 2008), while Ric-8B was shown to interact with Gα subunits *in vivo* in a nucleotide-dependent manner (Kerr et al. 2008), and both Ric-8A and Ric-8B were reported to amplify GPCR signaling *in vivo* (Von Dannecker, Mercadante, and Malnic 2005; Fenech et al. 2009).

GDP-Bound State Interactions:

The four families of mammalian proteins that contain GoLoco motifs, which possess a GDI functionality, are reviewed by Siderovski and Willard (Siderovski and Willard 2005).

Transition State Interactions:

The RGS family containing proteins that GAP Gα subunits preferentially interact with the transition state and are reviewed by Siderovski et al. (Siderovski and Willard 2005), Willars (Willars 2006), and Hurst and Hooks (Hurst and Hooks 2009). References for RhoGEF interactions are given in Results and in the Supplemental Discussion in Supplemental File 5. AKAP110 interaction with $G\alpha_{13}$ is reviewed in Niu et al (Niu et al. 2001).

GTP-Bound State Interactions:

Proteins that directly interact with G(12) subunits are reviewed by Kelly et al (Kelly, Casey, and Meigs 2007). The G(q) family subunits are reviewed by Hubbard and Hepler (Hubbard and Hepler 2006).

Nucleotide Independent Interactions:

Direct interactions between $G\alpha_{13}$ and integrins have recently been reported (Gong et al. 2010).

Depalmitoylation has also been reported: (Duncan and Gilman 1998) and (Tsutsumi et al. 2009).

REFERENCES

Duncan, Joseph A., and Alfred G. Gilman. 1998. A Cytoplasmic Acyl-Protein Thioesterase That Removes Palmitate from G Protein α Subunits and p21RAS. *The Journal of Biological Chemistry* 273 (June 19): 15830-15837.

Fenech, Claire, Lila Patrikainen, Daniel S. Kerr, Sylvie Grall, Zhenhui Liu, Fabienne Laugerette, Bettina Malnic, and Jean-Pierre Montmayeur. 2009. Ric-8A, a Gα Protein Guanine Nucleotide Exchange Factor Potentiates Taste Receptor Signaling. *Frontiers in Cellular Neuroscience* 3 (October 8): 1-9. doi:10.3389/neuro.03.011.2009.

Garcia-Marcos, Mikel, Pradipta Ghosh, and Marilyn G. Farquhar. 2009. GIV is a nonreceptor GEF for Gαi with a unique motif that regulates Akt signaling. *Proceedings of the National Academy of Sciences* 106, no. 9 (March 3): 3178-3183. doi:10.1073/pnas.0900294106.

Ghosh, Pradipta, Mikel Garcia-Marcos, Scott J. Bornheimer, and Marilyn G. Farquhar. 2008. Activation of G{alpha}i3 triggers cell migration via regulation of GIV. *J. Cell Biol.* 182, no. 2 (July 28): 381-393. doi:10.1083/jcb.200712066.

Gong, Haixia, Bo Shen, Panagiotis Flevaris, Christina Chow, Stephen C.-T. Lam, Tatyana A. Voyno-Yasenetskaya, Tohru Kozasa, and Xiaoping Du. 2010. G Protein Subunit G{alpha}13 Binds to Integrin {alpha}IIb{beta}3 and Mediates Integrin "Outside-In" Signaling. *Science* 327, no. 5963 (January 15): 340-343. doi:10.1126/science.1174779.

Hubbard, Katherine B., and John R. Hepler. 2006. Cell signalling diversity of the Gq[alpha] family of heterotrimeric G proteins. *Cellular Signalling* 18, no. 2 (February): 135-150. doi:10.1016/j.cellsig.2005.08.004.

Hurst, Jillian H., and Shelley B. Hooks. 2009. Regulator of G-protein signaling (RGS) proteins in cancer biology. *Biochemical Pharmacology* 78, no. 10 (November 15): 1289-1297. doi:10.1016/j.bcp.2009.06.028.

Juneja, Juhi, and Patrick J. Casey. 2009. Role of G12 proteins in oncogenesis and metastasis. *British Journal of Pharmacology* 158, no. 1 (September): 32-40.

Kelly, Patrick, Patrick J. Casey, and Thomas E. Meigs. 2007. Biologic Functions of the G12 Subfamily of Heterotrimeric G Proteins: Growth, Migration, and Metastasis†. *Biochemistry* 46, no. 23 (June 1): 6677-6687. doi:10.1021/bi700235f.

Kerr, Daniel S., Luiz Eduardo C. Von Dannecker, Marcela Davalos, Jussara S. Michaloski, and Bettina Malnic. 2008. Ric-8B interacts with G[alpha]olf and G[gamma]13 and co-localizes with G[alpha]olf, G[beta]1 and G[gamma]13 in the cilia of olfactory sensory neurons. *Molecular and Cellular Neuroscience* 38, no. 3 (July): 341-348. doi:10.1016/j.mcn.2008.03.006.

Le-Niculescu, Helen, Ingrid Niesman, Thierry Fischer, Luc De Vries, and Marilyn G. Farquhar. 2005. Identification and Characterization of GIV, a Novel Gαi/s -interacting Protein

Found on COPI, Endoplasmic Reticulum-Golgi Transport Vesicles. *The Journal of Biological Chemistry* 280, no. 23 (June 10): 22012-22020.

Niu, Jiaxin, Rita Vaiskunaite, Nobuchika Suzuki, Tohru Kozasa, Daniel W. Carr, Nickolai Dulin, and Tatyana A. Voyno-Yasenetskaya. 2001. Interaction of heterotrimeric G13 protein with an A-kinase-anchoring protein 110 (AKAP110) mediates cAMP-independent PKA activation. *Current Biology* 11, no. 21 (October 30): 1686-1690. doi:10.1016/S0960-9822(01)00530-9.

Siderovski, David P., and Francis S. Willard. 2005. The GAPs, GEFs, and GDIs of heterotrimeric G-protein alpha subunits. *International Journal of Biological Sciences* 1, no. 2: 51–66.

Tall, Gregory G., and Alfred G. Gilman. 2004. Purification and Functional Analysis of Ric-8A: A Guanine Nucleotide Exchange Factor for G-Protein [alpha] Subunits. In *Regulators of G-Protein Signaling, part B*, Volume 390:377-388. Academic Press. http://www.sciencedirect.com/science/article/B7CV2-4DK62YN-Y/2/c1f858ecb78e71b13b48ceca948309ee.

———. 2005. Resistance to inhibitors of cholinesterase 8A catalyzes release of Gαi-GTP and nuclear mitotic apparatus protein (NuMA) from NuMA/LGN/Gαi-GDP complexes. *Proceedings of the National Academy of Sciences of the United States of America* 102, no. 46 (November 15): 16584-16589. doi:10.1073/pnas.0508306102.

Tall, Gregory G., Andrejs M. Krumins, and Alfred G. Gilman. 2003. Mammalian Ric-8A (Synembryn) Is a Heterotrimeric Gα Protein Guanine Nucleotide Exchange Factor. *The Journal of Biological Chemistry* 278, no. 10 (March 7): 8356-8362.

Thomas, Celestine J., Gregory G. Tall, Anirban Adhikari, and Stephen R Sprang. 2008. Ric-8A Catalyzes Guanine Nucleotide Exchange on Gαi1 Bound to the GPR/GoLoco Exchange Inhibitor AGS3. *The Journal of Biological Chemistry* 283, no. 34 (August 22): 23150-23160.

Tsutsumi, Ryouhei, Yuko Fukata, Jun Noritake, Tsuyoshi Iwanaga, Franck Perez, and Masaki Fukata. 2009. Identification of G Protein {alpha} Subunit-Palmitoylating Enzyme. *Mol. Cell. Biol.* 29, no. 2 (January 15): 435-447. doi:10.1128/MCB.01144-08.

Von Dannecker, Luiz Eduardo C., Adriana F. Mercadante, and Bettina Malnic. 2005. Ric-8B, an Olfactory Putative GTP Exchange Factor, Amplifies Signal Transduction through the Olfactory-Specific G-Protein G{alpha}olf. *J. Neurosci.* 25, no. 15 (April 13): 3793-3800. doi:10.1523/JNEUROSCI.4595-04.2005.

Willars, Gary B. 2006. Mammalian RGS proteins: Multifunctional regulators of cellular signalling. *Seminars in Cell & Developmental Biology* 17, no. 3 (June): 363-376. doi:10.1016/j.semcdb.2006.03.005.
