# Supplementary material for: Evolution of a Signaling Nexus Constrained by Protein Interfaces and Conformational States
Source: PLoS Comput Biol. 2010 Oct 14;6(10):e1000962. doi: 10.1371/journal.pcbi.1000962 (PMC2954821; doi:10.1371/journal.pcbi.1000962)
Supplement: Text S3 — Comparison of evolutionary trace and class-distinctive site analyses. (0.09 MB PDF) [file pcbi.1000962.s008.pdf]

Comparison of Class-Distinctive and Evolutionary Trace Sites

Level #	Invariant	G(i)-D	G(q)-D	G(s)-D	G(12)-D	Total Invariant + Distinctive Sites	Total New ET Sites @ Level
1	76					76	76
2	9			7	8	24	43
3	2	3	2	1		8	13
4							
5							
6	4	2			7	13	46
7	1	1		1	2	5	18
8	6	3	3	3	1	16	75
9	1					1	13
10	3		3		1	7	24
Class-D Not Found by ET	2	4	2	4		12	
**TOTAL**	**104**	**13**	**10**	**16**	**19**		**308**

Many, but not all, class-distinctive sites are also identified by the Evolutionary Trace (ET) method. Only 76 of the 104 invariant sites spanning the section of the MSA covered by the crystallographic structure were identified at the equivalent partition level (level 1) of the ET method (Table S5). A number of invariant sites, however, were found at other partition levels, with 2 invariant sites not identified at all by ET. These differences can be accounted for by the different amino acid alphabets used in the two analyses. Our class-distinctive analysis used a reduced amino acid alphabet (e.g. R=K; see Materials and Methods), whereas ET did not (e.g. R≠K). The lowest partition level with more than 1 group, level 2, broke the sequences up into three groups: G(i) and G(q) in the first group; G(s) in the second group; and G(12) in the third group. Slightly less than half of the G(s)-D and G(12)-D sites were identified at this partition level, with most of the remaining G(s)-D and G(12)-D sites selected at other partition levels. Partition level 3, with G(i) and G(q) sequences in separate groups, most closely corresponds with the class-distinctive analysis. Several G(i)-D and G(q)-D sites are detected at this level. Differences in the amino acid alphabet used in the two methods accounts for some of the dispersion of the class-distinctive sites across the different partition levels of ET. It also is likely to be a significant factor in the 10 (of 59 total) class-distinctive sites not identified at all by ET. But allowing subclass variation within the distinctive class, as we did, also likely contributes to the dispersion of class-distinctive sites across different ET partition levels. The comparison of the two methods suggests the class-distinctive analysis is, in some sense, a projection of class-distinctive sites from all ET partition levels onto a single level.

Class-distinctive and invariant sites are not the only sites identified at each partition level, and may not even be the majority of the sites at that partition level. Additional sites identified by ET but not by the class-distinctive analysis include conserved insertions, and sites with a more complex evolutionary history. For example, at partition level 2, G(i)=G(q) but G(s) and G(12) are different. At two such sites, G(s)=G(12) and at four such sites, G(s) is different from G(12) , none of which fit our criterion for a class-distinctive site. In addition, thirteen sites at partition level 2 are from class-specific insertions, and 24 correspond to invariant or class-distinctive residues. Hence, the class-distinctive sites comprise a subset of all sites contributing to class-specific function, but it is a subset likely to be critical to the establishment of class-specific function in Gα evolution.
