# Supplementary material for: Evolution of a Signaling Nexus Constrained by Protein Interfaces and Conformational States
Source: PLoS Comput Biol. 2010 Oct 14;6(10):e1000962. doi: 10.1371/journal.pcbi.1000962 (PMC2954821; doi:10.1371/journal.pcbi.1000962)
Supplement: Text S4 — Additional discussion of RhoGEF and G(12) families. (0.08 MB PDF) [file pcbi.1000962.s009.pdf]

In humans, there are 2 additional members besides p115RhoGEF in the RGS-box-containing RhoGEF family, leukemia-associated RhoGEF (LARG) and PDZ-RhoGEF. LARG RhoGEF activity was stimulated by interactions with $G\alpha_{13}$ but, in contrast with p115RhoGEF, LARG GEF activity could also be mediated by $G\alpha_{12}$ (Suzuki et al. 2003). PDZ-RhoGEF activation of RhoA has also been shown to be mediated by both $G\alpha_{12}$ and $G\alpha_{13}$ (Fukuhara et al. 1999). Stimulation of RhoA activation by G(12) family members did show additional subtype functional variation, as RhoGEF activity was dependent upon the phosphorylation state of LARG. $G\alpha_{12}$-mediated activation of RhoA was effectively stimulated by tyrosine phosphorylated LARG whereas activation of RhoA by nonphosphorylated LARG was mediated through $G\alpha_{13}$ but not $G\alpha_{12}$ (Suzuki et al. 2003). Thus it appears that gene duplications both within this RhoGEF family and within the G(12) family have led to the emergence of paired interactions with a variety of functional outcomes.

As already noted, G(12) is the only $G\alpha$ class that had distinctive sites in switch I, sites that make direct contact to a novel structural element that is a GAP for both $G\alpha_{12}$ and $G\alpha_{13}$. Both family members possessed the ∂ amino acid values in switch I, consistent with the shared functional response. Only two $G\alpha$ classes, G(12) and G(s), had distinctive sites in switch II. Two G(12) sites in switch II formed contacts with the RGS-like box of the G(12)-specific interactor p115RhoGEF. Subtype variations in sequence was evident at three of the four G(12) distinctive sites in switch II, including one of the sites that directly contacted p115RhoGEF. While it is reasonable to hypothesize that subtype-specific sequence variations at G(12) sites 11 and 12 and the two other sites in switch II potentially affect the subtype-specific RhoGEF activity reviewed above, it is also possible that other effectors evolved to use these residues for subtype discrimination given their presence in this important functional region. Our analysis

suggested several G(12) sites in switches I and II are constrained in evolution by interactions with p115RhoGEF or other homologs in this RhoGEF family and may contribute to subtype-specific functionality, in addition to class-specific functionality.

Understanding the structural elements, both on Gα and on RhoGEF, driving the varied functional outcomes in the interactions between G(12) subunits and the three members of the RhoGEF family is critical for understanding the role of these GPCR signaling pathways. But of equal interest is the manner in which this complexity of interactions evolved. There is a single G(12) subunit and a single RhoGEF member in invertebrates, extant proteins which shared common ancestors with the mammalian proteins of these two families. Surprisingly, the single G(12) subunit in invertebrates contains the ∂ amino acids in G(12) sites in switch II. After the gene duplication giving rise to the two G(12) subunits present in vertebrates, these ∂ amino acids revert back to the **η** amino acids in $G\alpha_{13}$ – ∂ to **η** rather than the canonical **η** to ∂ – but remain the ∂ amino acids in $G\alpha_{12}$. One result of this interesting evolutionary step in switch II is that the mammalian $G\alpha_{12}$ subunit contains the switch II found in invertebrates. In mammals, $G\alpha_{12}$ stimulates RhoGEF activity of PDZ-RhoGEF and phosphorylated LARG, but does not stimulate the RhoGEF activity of p115RhoGEF. These varied functional outcomes drive the intriguing question, which functional outcome reflects the primordial function evident in invertebrates?

REFERENCES

Fukuhara, Shigetomo, Cristina Murga, Muriel Zohar, Tadashi Igishi, and J. Silvio Gutkind. 1999. A Novel PDZ Domain Containing Guanine Nucleotide Exchange Factor Links Heterotrimeric G Proteins to Rho. *J. Biol. Chem.* 274, no. 9 (February 26): 5868-5879. doi:10.1074/jbc.274.9.5868.

Suzuki, Nobuchika, Susumu Nakamura, Hiroyuki Mano, and Tohru Kozasa. 2003. Gα12 activates Rho GTPase through tyrosine-phosphorylated leukemia-associated RhoGEF. *Proceedings of the National Academy of Sciences of the United States of America* 100, no. 2 (January 21): 733-738. doi:10.1073/pnas.0234057100.
